# Simultaneous analysis of pesticides and mycotoxins in primary processed foods: The case of bee pollen

**DOI:** 10.1016/j.heliyon.2024.e33512

**Published:** 2024-06-26

**Authors:** Maria Antonietta Carrera, José Antonio Martinez Martinez, María Dolores Hernando, Amadeo R. Fernández-Alba

**Affiliations:** a Department of Desertification and Geo-ecology, Experimental Station of Arid Zones, CSIC, Ctra. Sacramento s/n, La Cañada de San Urbano, 04120, Almería, Spain; b European Union Reference Laboratory for Pesticide Residues in Fruit & Vegetables Agrifood Campus of International Excellence (ceiA3), Department of Chemistry and Physics, University of Almeria, Ctra. Sacramento s/n, La Cañada de San Urbano, 04120, Almería, Spain

**Keywords:** Primary processed foods, Bee pollen, Pesticides, Mycotoxins, LC-MS/MS

## Abstract

Primary Processed Foods are a class of food items that are ready for consumption after minimal processing in the supply chain. These products are ubiquitous in our daily diet, but so far a limited number of studies dealt with the optimization of quality control methods to check their content of contaminants. Among primary processed foods, bee pollen is a nutritionally acclaimed food supplement, whose contamination with pesticides and mycotoxins has been largely proven. For this reason, the present study aimed at optimizing for the first time a comprehensive LC-MS/MS method capable of analyzing 282 pesticides and 8 mycotoxins in bee pollen. To obtain a suitable method, two extraction procedures (QuEChERS and Accelerated Solvent Extraction), as well as different chromatographic gradients and columns, were tested. The optimized methodology, comprehending an extraction based on semi-automated QuEChERS, and an analytical method including inert LC column technology, was validated and applied to a sample set of 34 bee pollens. The analyzed samples collectively showed the presence of 41 pesticides and 1 mycotoxin.

## Highlights

• Optimized QuEChERS extraction for pesticides and mycotoxins from bee pollen.
• QuEChERS and ASE showed comparable extraction performances.
• Use of an 11-min chromatographic gradient and an inert column for LC-MS/MS analysis.
• Method validated for the analysis of 282 pesticides and 9 mycotoxins in bee pollen.
• Detection of ochratoxin A and 41 pesticides in the analyzed samples.

## 1 Introduction

Primary Processed Foods (PPF) are those products – mostly raw materials - that undergo minimal transformation in the supply chain, in order to be easily transported and ready to be sold, eaten, or used by secondary manufacturers [1]. Common product treatments employed in primary food processing are drying, milling, shelling etc., and some of the most representative products obtained by the primary food industry are plant-based items like flours, sugars, vegetable oils, and cocoa [2]. Overall, a great variety of food items and supplements that we consume everyday are part of the PPF category.

An increasingly popular PPF, whose market is experiencing an exponential growth trend, is bee pollen [3]. This beekeeping product is generally collected from the hive and immediately dried at temperatures not higher than 42 °C [4]. This allows bee pollen to have a longer shelf-life without requiring refrigeration. The increasing popularity of bee pollen as food supplement is due to its unique chemical composition, rich in nutrients and bioactive compounds, and to its proven therapeutic properties [5]. Due to its characteristics, bee pollen is also attracting increasing interest in the feed market for its potential applications as a nutritional supplement for livestock and as feed for bumblebees - traded on an industrial scale for pollination of crops - or for edible insects used as a protein source [6]. However, several studies have shown the presence of contaminants of known toxicity in bee pollen, sometimes reaching potentially dangerous concentrations [7]. Particularly alarming contaminants found in bee pollen are mycotoxins, toxic metabolites produced by fungi in favorable environmental conditions [8], and pesticides, commonly employed for pest control, which pose a potential health hazard for consumers [[9], [10], [11]]. The contamination of bee pollen with such contaminants can have several sources. Regarding pesticides, the exposure of bees to veterinary treatments employed in beekeeping against bee pests, and to plant protection compounds found in the environment where they forage (due to recent application or to their persistence), can cause the transfer of such chemicals to bee products [12]. On the other hand, mycotoxins contamination can occur at any stage of the production chain. Due to optimal moisture content, water activity, and pH, pollen often presents an ideal environment for the proliferation of fungi, which can produce mycotoxins if favorable conditions occur during bee pollen's handling or storage. In addition to production process and human hygiene practices, mycotoxins contamination can also be sometimes caused by the interaction between bees and flowers infected with fungal spores. This variety of factors makes the control of such contaminants in bee pollen a very complex issue [13].

Preceding studies to this current work, conducted on a sample set of eighty bee pollen samples from different countries, revealed the presence of four different mycotoxins (aflatoxin B1, deoxynivalenol, zearalenone, and ochratoxin A) and 77 different pesticides, including plant protection chemicals and veterinary treatments, in the analyzed samples. The risk assessment performed in these studies showed that mycotoxins reached potentially hazardous concentrations for consumers in a high percentage of the analyzed cases, while pesticides seemed not to pose a concrete health risk. However, in both cases the risk evaluation had to be performed through a series of approximations due to the lack of data on bee pollen average consumption, and of specific limits for both types of contaminants in this bee product [14,15].

The findings of these studies are even more concerning when we consider that the climatic and environmental changes that our planet has experienced in recent decades could worsen this scenario. In fact, food contamination is expected to be exacerbated by the effects of climate change. Particularly, the changes in temperature, humidity, precipitations and CO_2_ levels can have an impact on fungi proliferation and on mycotoxins production, but also on the geographical distribution of agricultural pests, possibly causing an increase in pesticides usage [16,17]. Therefore, it has become essential to provide specific analytical methods for the analysis of such concerning contaminants in PPF like bee pollen.

Mass spectrometry-based methods for the simultaneous analysis of different classes of contaminants, like pesticides and mycotoxins, represent a reliable option for the quality control of food and feed items. The optimization of such comprehensive methods often presents the challenge of achieving an adequate extraction and a good sensitivity for all the compounds included in the analysis, but at the same time presents the advantage of being a faster and cheaper alternative to the classical methods [18,19]. In recent years, a variety of multi-contaminants methods have been developed for a variety of food matrices, including fruits, vegetables, dairy and infant products. A very limited number of studies dealt with the simultaneous detection of mycotoxins and pesticides in a few PPF (e.g. cereals and nuts), but, despite its demonstrated content of hazardous contaminants, no specific method was developed for bee pollen [20,21].

For this reason, the main aim of this work was to provide a new fast, sensitive, and reliable tool for the simultaneous analysis of two different classes of contaminants in bee pollen. Specifically, a LC-MS/MS method was developed for the targeted analysis of a set of 8 mycotoxins and 282 pesticides, including both emerging and known compounds. To allow the detection of these contaminants, often present at traces level, a thorough process of extraction optimization was conducted by comparing a semi-automated QuEChERS approach, with a fully automated Accelerated Solvent Extraction (ASE) methodology. For a further improvement of the analytical performance, two chromatographic gradients of different length, and two chromatographic columns with different characteristics were compared. The obtained analytical method was validated and employed for the analysis of 34 bee pollen samples collected from Slovenia and Spain.

## 2 Materials and methods

### 2.1 Reagents and materials

All high purity (≥98 %) pesticide standards were obtained from Sigma-Aldrich (Steinheim, Germany) and LGC (Teddington, UK), and stored at −30 °C. Individual pesticide stock solutions (1000–2000 mg/L) were prepared in acetonitrile and stored in amber glass vials in the dark at −20 °C. Individual standard solutions, used for optimization, and standard-mix solutions, used for calibration, were prepared from stock standards. The pesticides analyzed, listed in Table S2, were selected to include veterinary treatments used in beekeeping, plant protection treatments previously detected in beekeeping products by EU monitoring programs, and pesticides included in the EU monitoring priority list [22].

Mycotoxin standards (aflatoxin B1, deoxynivalenol, zearalenone, ochratoxin A, enniatin A1, enniatin B1, apicidin, sterigmatocystin, ochratoxin A-^13^C20) were purchased from Sigma-Aldrich (Steinheim, Germany) and stored following the producer's recommendation. Individual pesticide stock solutions (2–1000 mg/L) were prepared in 80 % acetonitrile, with 20 % water and 0.1 % formic acid, or methanol according to their stability in these solvents, and stored in amber glass vials in the dark at −20 °C. Individual standard solutions, used for optimization, and standard-mix solutions, used for calibration, were prepared from stock standards. The mycotoxins included in the method were chosen to include compounds previously detected in bee pollen, and mycotoxins of emerging concern.

For the QuEChERS extraction, QuEChERS salts (anhydrous magnesium sulphate, sodium chloride, sodium citrate dibasic sesquihydrate and sodium citrate tribasic dihydrate), supplied by Sigma-Aldrich (Steinheim, Germany), and μSPE-GCQuE1-45 cartridges (μSPE cartridges for the PAL® system containing a 20/12/12/1 ratio of anhydrous MgSO_4_/PSA/C18/CarbonX, respectively), supplied by CTC Analytics (Zwingen, Switzerland), were used.

Accelerated Solvent Extraction was conducted by employing specific diatomaceous earth and cellulose filters for ASE applications, both purchased from ThermoFisher Scientific (Waltham, USA).

The solvents used were: water Optima™ LC/MS grade (Fisher Chemical (Geel, Belgium)), acetonitrile (ACN) HPLC grade (purity ≥99.9 %) and ACN LC/MS grade (purity ≥99.9 %) from Honeywell/Riedel-de Haën (Seelze, Germany), formic acid (98 %) from Fluka Analytical (Steinheim, Germany), ammonium formate from Sigma-Aldrich (Steinheim, Germany) and methanol from Fluka Analytical (Steinheim, Germany).

### 2.2 Sampling

For this study, 34 samples of pollen loads were purchased from Slovenia and Spain. Slovenian bee pollen samples (n = 20) were obtained from the Slovenian Beekeepers’ Association in the summer of 2023. Samples were collected from 6 different regions, and stored in their fresh form at −20 °C before analysis. Spanish bee pollen samples (n = 14) were purchased from herbal stores, grocery stores and mainly from e-Commerce providers. They were bought dry and stored at room temperature before analysis. Both unprocessed (fresh) and processed (dry) bee pollen samples were included in this study in order to qualitatively compare their degree of contamination with moisture dependent compounds, like mycotoxins. Following collection, all samples were listed, and assigned an ID. Before analysis, bee pollen samples were milled and subjected to moisture content determination employing a halogen Moisture Analyzer HE73 by Mettler Toledo (Columbus, USA). More information on the sample set is reported in Table S1 of the supplementary material.

### 2.3 Sample treatment

In this study two extraction methods, respectively employing QuEChERS and Accelerated Solvent Extraction, were tested and compared for the extraction of pesticides and mycotoxins from bee pollen.

1 QuEChERS with μSPE clean up

For this procedure, samples were extracted using the QuEChERS method. According to the procedure previously optimized for the analysis of pesticides in bee pollen [15], 2 g of sample were weighed in a 50-mL PTFE centrifuge tube and 2 mL of H_2_O were added. The tubes were vortexed manually for 3 s and 4 mL of acetonitrile were added. The samples were then shaken in an axial agitator (Agytax, Agytax Lab, Madrid, Spain) for 4 min and then 1.3 g of QuEChERS salts were added. The tubes were automatically shaken again for 4 min and then centrifuged at 3.200 RCF for 5 min. To obtain the precipitation of lipids and proteins, 4 mL of the supernatants were transferred to a 15-mL PTFE tube and subjected to a 3 min freeze in dry ice. Then, an automated clean-up procedure was performed with μSPE cartridges using a PAL® RTC X-Y-Z auto sampler (CTC analytics, Zwingen, Switzerland). For the automated process, 200 μL of raw extract sample, obtained after the freezing out, were mixed with 50 μL of acetonitrile. Then, the same workflow previously optimized [23] for μSPE automated clean-up was used. First, the cartridges were preconditioned with 100 μL acetonitrile prior to sample loading. Then, 200 μL of each sample raw extract was loaded into the cartridge at 5 μL/s and the clean extract was collected in a 2-mL vial with a pre-cut septum cap. Cartridges were eluted with 100 μL acetonitrile (5 % formic acid) and the clean extract was collected into a 2-mL vial with a pre-cut septum cap.

2 Accelerated solvent extraction (ASE)

Accelerated Solvent Extraction was performed employing an EXTREVA ASE (ThermoFisher Scientific), equipped with 22-mL stainless steel extraction cells. Sample extraction was realized following the manufacturer's instructions. 3 g of bee pollen were weighed in a 22 mL-steel cell, and on top of it, 3.8 g of diatomaceous earth, were added. Two cellulose filters were inserted on both sides of the extraction cell, which was then sealed and put in the ASE system. The extraction was performed by setting the instrument parameters as follows: oven temperature was 45 °C, and the applied pressure 1500 psi; cell fill volume was set at 50 %, the solvent flow rate was 0.5 mL/min, and the extraction time was 8 min. Acetonitrile was used as extraction solvent and nitrogen was employed as purging gas (purging time: 30 s, gas flow rate: 10 mL/min). A total extraction volume of 20 mL was estimated by the ASE system.

After the extraction process was completed, 4 mL of extract were transferred to a 15 mL- PTFE tube and subjected to a 3 min freeze in dry ice. The supernatant was then transferred to a 4 mL-vial.

The extracts obtained by employing both extraction approaches were diluted with Optima™ water containing dimethoate-d6 as injection standard prior to their analysis. Vials for LC analysis were prepared considering the same proportion of matrix:solvent in both the analyzed cases. Then the extracts were analyzed by LC-MS in full scan mode, and the results obtained were compared in terms of extract cleanness (carried out by comparing the height of the baselines in the obtained total ion chromatograms). Furthermore, the analytes recoveries obtained with both extraction techniques were evaluated by fortifying a bee pollen sample with 10 μg/kg of standard mix. The fortified sample was extracted by QuEChERS and ASE in triplicate and the extracts obtained were analyzed by targeted LC-MS/MS.

Once the best extraction procedure was selected, the purchased bee pollen samples were extracted and analyzed. For them, procedural internal standards were used as surrogate standards to control the extraction performance: dichlorvos-d6, malathion-d10, carbendazim-d3, and ochratoxin A-^13^C20. The calibration curve was obtained by performing the selected extraction procedure on a blank matrix and adding aliquots of the standard mix.

### 2.4 Analysis by LC-QqQ-MS/MS

A Sciex Exion HPLC coupled to a Sciex 6500+ TripleQuand-LC-MS/MS from Sciex was used for the analysis. Chromatographic separation was tested on two different columns: a Zorbax Eclipse Plus C8 of 1.8 μm × 2.1 mm × 100 mm (Agilent Technologies – Santa Clara, USA), and a Raptor Inert ARC-18 2.7 μm × 2.1 mm × 100 mm (Restek – Bellefonte, USA), both kept at a stable temperature of 40 °C. Mobile phase A was 98 % water and 2 % methanol, while mobile phase B was 98 % methanol and 2 % water. Both mobile phases contained 5 mM ammonium formate and 0.1 % formic acid. In order to optimize the sensitivity of the analytical method, two gradient programs of different length were tested (Table 1). The mobile phase flow was 0.30 mL/min, and the injection volume was 2.5 μL.

**Table 1:** Chromatographic gradients.

11-min gradient		15-mingradient	
Time (min)	**% Phase A**	**Time (min)**	**% Phase A**
**0**	100	0	100
**1**	100	1	100
**2**	70	2	70
**3**	50	3	50
**11**	0	15	0
**14**	0	17	0
**14.1**	100	17.1	100
**17**	100	18	100

Mass spectrometry analysis was performed in Multiple Reaction Monitoring (MRM) ion mode, with both positive and negative ionizations obtained by employing an ESI source switching polarity. The ionization settings used were: curtain gas, 25 psi; ion source gas 1, 40 psi; ion source gas 2, 40 psi; and temperature, 300 °C. The ion spray voltages were set at 5500 and −4500 V respectively in positive and negative ionization mode. Nitrogen was used as the nebulizer gas and collision gas. The compounds list and the associated analytical parameters are reported in Table S2.

Both columns and both chromatographic methods were tested on blank bee pollen extracts spiked with 1 μg/kg, 5 μg/kg and 10 μg/kg of standards mix. Parameters like the distribution of the analytes along the chromatography, the dwell time associated to each one of them, and the instrumental sensitivity obtained for each compound were evaluated to choose the best column and the best gradient.

### 2.5 Method validation

The identification of pesticides and mycotoxins, and the validation of the analytical method were based on the criteria established in the SANTE document [24].

For the identification of the target compounds, 2 precursor-ion/product-ion transitions were chosen for each compound to obtain perfect peak overlap, the same peak shape, and a signal-to-noise ratio greater than 3. Furthermore, the ion ratio (relationship between the abundance of the selected transitions) of the sample extracts had to be within ±30 % (relative) of the average of the calibration standards of the same sequence, and the retention time tolerance was ±0.1 min with the standard.

The procedure followed for the validation of the method and the performance for the quantification of pesticides has been described in detail by Refs. [25,26]. Recovery of the extraction method was determined at two concentration levels, LOQ and 10*LOQ μg/kg. Five replicates were carried out at each concentration level to verify recovery. Repeatability (RSDr) and reproducibility (RSDwR) within the laboratory were tested for spike levels LOQ and 10*LOQ μg/kg over 1 and 5 days, respectively. LOQ was defined as the lowest spike level that met the identification and method performance criteria for recovery (recovery in the range of 70–120 %) and precision (RSD <20 %). Linearity was tested by evaluating the signal responses of target analytes from matrix-matched calibration solutions prepared by spiking blank extracts at five concentration levels, from 0.25 to 100 μg/kg. Bee pollen Matrix Effect (ME) was studied by comparing the slope of the calibration curve in solvent standards with the slope of the matrix-matched calibration curve. Due to the heterogeneity of bee pollen samples, whose chemical characteristics depend on floral, geographical origin etc, two matrix-matched calibration curves were prepared using two bee pollen samples differing in color, moisture content and country of origin. This was done in order to evaluate the suitability of a quantification process based on a single calibration curve for all the analyzed bee pollen samples.

The expanded measurement uncertainty (U′) was also calculated for all the analytes included in the method to provide the range around the experimental results within which the true values are expected to lie within a defined probability. First, RSDwR and the bias component (estimated from recoveries) were used to evaluate the measurement uncertainty (U), then the expanded uncertainty (U′) was calculated by multiplying the measurement uncertainty by a coverage factor k = 2. Furthermore, the expanded measurement uncertainty for groups of compounds with the same LOQ was calculated (at LOQ and 10*LOQ), in order to have a more global vision of the method's uncertainty.

### 2.6 Statistical analysis

To guarantee the reliability of the results obtained from sample analysis, this work adhered to the standard procedure utilized by routine analysis laboratories according to ISO 17025 and SANTE Guidelines. This usually involves conducting triplicate analysis only when the detected contaminants exceed or are very close to the MRL (for pesticides) or the maximum level (for mycotoxins) set by the EU for that specific compound in the food product under analysis. However, due to lack of specific limits for both mycotoxins and pesticides in bee pollen, an approximation had to be performed when choosing the reference limit. Specifically, in the case of pesticides, triplicate analyses were performed only when the detected concentration exceeded 10 μg/kg, a level at which the pesticide concentration is deemed insignificant for compliance in products consumed in much larger quantities than bee pollen, such as tomatoes. The same protocol was applied when the mycotoxin concentration in a sample surpassed the EU-established limit for that compound in processed cereals available on the market (3 μg/kg for ochratoxin A) [27,28].

The standard deviation calculated for the results exceeding the reference limits was reported in Table S5 of supplementary material.

## 3 Results and discussion

### 3.1 Sample treatment

Due to the complexity of bee pollen's chemical composition, obtaining clean extracts from this matrix can be a challenging process. Nevertheless, this is an essential requirement for contaminants analysis, where matrix effect, ion suppression and interferences constantly need to be minimized. In this study, two different extraction approaches - QuEChERS and ASE - were tested on bee pollen and evaluated in order to find the most suitable procedure for the simultaneous extraction of pesticides and mycotoxins from this matrix. QuEChERS is a technique mostly used for pesticides analysis, but thanks to its versatility it has also been employed for the simultaneous extraction of different classes of contaminants, including mycotoxins, as reported by previous studies [29]. On the other hand, Accelerated Solvent Extraction is an automated high-pressure, high-temperature approach that can be employed for the extraction of different classes of contaminants, including pesticides and mycotoxins [30]. A limited number of past studies specifically tested the ASE methodology on beekeeping products, showing promising results [31].

**Fig. 1:**
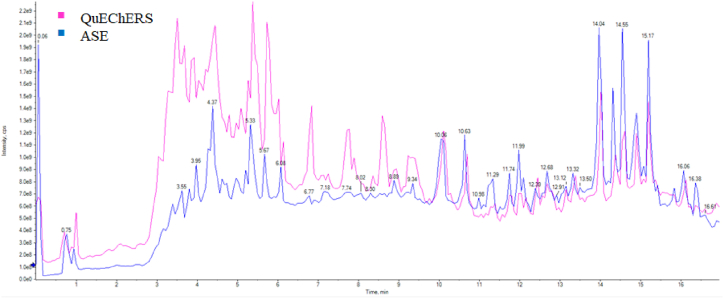
TICs obtained from the Full Scan analysis of a blank bee pollen sample subjected to QuEChERS and ASE protocols.

To compare the performances of the employed extraction techniques, the extracts obtained from a blank pollen sample with both QuEChERS and ASE methodologies were analyzed by LC-MS in full scan mode in order to evaluate the extract cleanness, by comparing the Total Ion Chromatograms (TICs) (Fig. 1).

From the overlapped visualization of the TICs it is possible to observe that the baseline obtained with the ASE methodology is mostly lower than that obtained with the QuEChERS extraction, especially in the 3–6 min time frame, where the most polar compounds are eluted. This is likely an effect of sample hydration, a step that is generally included in the QuEChERS methodology when this is applied to matrices characterized by a low content of water (less than 75 %). Hydration has the main objective of making the matrix's pore more accessible to the extraction solvent. However, it has been observed that despite enhancing the extraction performance, the hydration step can cause the co-extraction of several polar compounds from the matrix, which can interfere with the analytes detection [32,33]. The employment of high temperatures in the ASE methodology results in an increase in analytes solubility, and a decrease in solvent viscosity, which improve analytes diffusion into the solvent, hence allowing to avoid sample hydration. This ultimately causes a lower input of matrix interferers in the instrument, which has the double effect of reducing ion suppression, and minimizing the ordinary maintenance of the instrument.

To obtain more data on the compared efficiency of QuEChERS and ASE extraction techniques, the recovery of 291 compounds, including pesticides and mycotoxins, at 10 μg/kg was tested for both. Comparable results were obtained, since both techniques showed good and reproducible recovery values for all the compounds of interest (Table S4).

However, when comparing all the characteristics of both extraction techniques for the selection of the best strategy (Table 2), a strong limitation for the ASE methodology was observed. In fact, the EXTREVA ASE system requires the usage of greater solvent volumes (20 mL against 4 mL used in the QuEChERS methodology), which leads to excessive analytes dilution. The employment of such volumes is dictated by sample cell size, which in our case was of 22 mL. To overcome this problem the purchase of smaller extraction cells would be necessary.

**Table 2:** Summarized characteristics and results of QuEChERS and ASE extraction strategies applied to bee pollen.

	Automated	Extraction duration	Sample hydration	Volume of solvent	Recovery range	RSD range
QuEChERS	Partially	User dependent	Yes	4 mL	75–120 %	2.8–15.0 %
ASE	Fully	17 min	No	20 mL	70–120 %	2.1–14.9 %

### 3.2 Analysis by LC-QqQ-MS/MS

To improve the sensitivity of the analytical LC-MS/MS method, two chromatographic columns (Zorbax C8 and Raptor Inert C18) and two gradients (11-min and 15-min) were compared and evaluated according to different parameters.

**Fig. 2:**
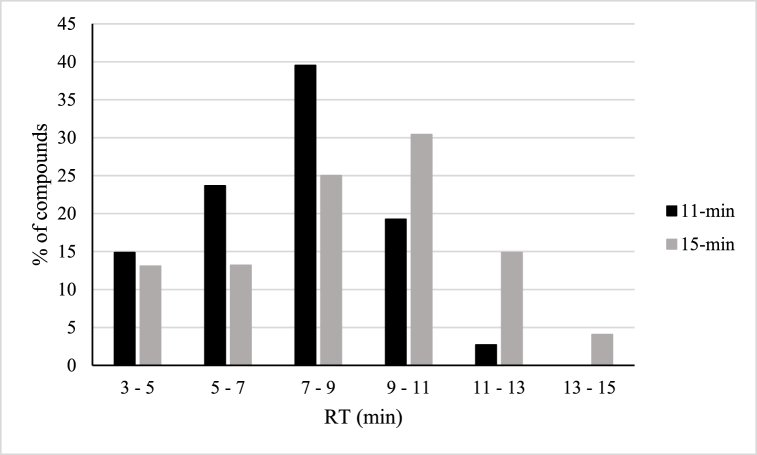
Distribution of the analytes along the chromatography obtained by employing the 11-min and the 15-min gradients.

First, a pollen blank extract spiked with 10 μg/kg of standard mix was injected with both gradients and the distributions of the analytes along the chromatography were compared. As shown in Fig. 2, the 15-min gradient led to a more homogeneous distribution of the compounds included in the method, reducing the number of compounds eluted in particularly “crowded” time frames.

Due to the large number of compounds included in the method, the employment of a 15-min gradient also led to a variation in the dwell time automatically calculated by the mass spectrometer for each compound. It was observed that, while using the 11-min gradient, 49 % of the analyzed compounds had the lowest applicable dwell time (3 ms). When the analysis was performed with the 15-min gradient, this percentage decreased to the 36 %. As is known, an increase in the dwell time can contribute to the improvement of analytical sensitivity.

**Fig. 3:**
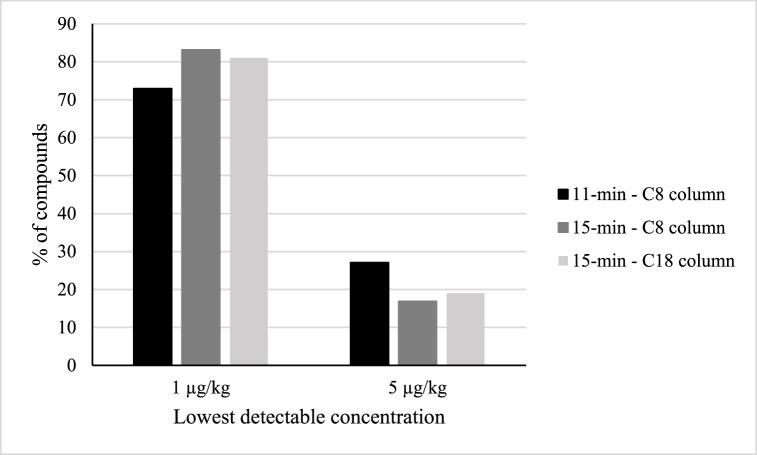
Lowest detectable concentrations obtained using the tested gradients (11-min and 15-min-) and chromatographic columns (C8: Zorbax – C18: Restek).

To furtherly verify the improvement produced by the extension of the gradient, pollen blank extracts respectively spiked with 1 and 5 μg/kg standard mix were injected with both chromatographic gradients. This was done with the aim of visualizing possible variations in the sensitivity of the method. Also this test corroborated the hypothesis that the longer chromatography was actually improving the analytical sensitivity. As shown in Fig. 3, a larger number of compounds could be detected at a concentration of 1 μg/kg when switching from the 11-min to the 15-min gradient. Clearly, the sensitivity could have furtherly been improved by using an even longer gradient. However, taking into account the desired LOQs and the overall improvement, this approach seemed the best compromise between a fast and a sensitive analytical method.

**Fig. 4:**
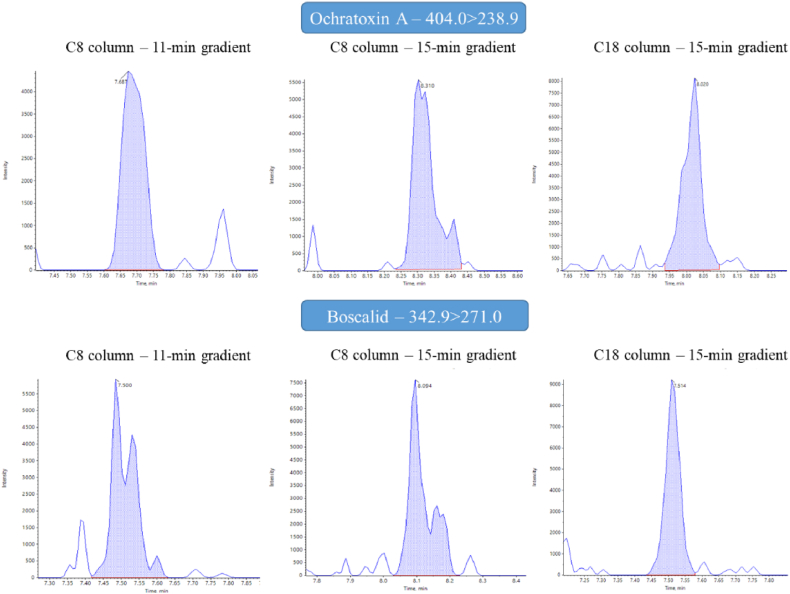
Improvement of peak shape achieved with the optimization of chromatographic gradient (11-min and 15-min) and column (C8: Zorbax – C18: Restek). Example reporting one mycotoxin and one pesticide included in the method.

Once the longer gradient was selected, the subsequent parameter to be tested was the type of column used for the analytes separation. The two columns compared in this study were a Zorbax Eclipse Plus C8 (1.8 μm × 2.1 mm × 100 mm, Agilent Technologies), widely employed in multiresidues analysis, and a Raptor Inert ARC-18 (2.7 μm × 2.1 mm × 100 mm, Restek), whose inert characteristics are designed to reduce the interactions between steel and metal-sensitive compounds. Also for this test, blank pollen extracts respectively spiked with 1 and 5 μg/kg standard mix were injected in both columns, and the lowest detectable concentration was evaluated for each analyte. As reported in Fig. 3, no substantial differences were revealed by this test, since almost the same number of compounds could be detected at a concentration of 1 μg/kg with both columns. However, by looking at peaks obtained for about 15 % of analytes at 1 μg/kg, it was possible to notice a visible improvement in the peak shape (Fig. 4) when using the Raptor Inert column. This particular feature can be sometimes decisive for the identification of the analytes in real samples.

### 3.3 Selection of the best methodology

Given all the previous considerations, the semi-automated QuEChERS procedure with μSPE clean up was chosen as the best extraction methodology for bee pollen. Furthermore, the 15-min chromatographic gradient and the Restek column were selected for the analysis of our target compounds in bee pollen samples. The described analytical method was validated and then applied to the analysis of real samples.

### 3.4 Method validation

For the optimized LC-MS/MS method the LOQ was at 1–5 μg/kg for all compounds tested in bee pollen (except deoxynivalenol, whose LOQ was set at 100 μg/kg), since these were the lowest spike level that met the identification and method performance criteria reported in Document SANTE/11312/2021 V2. Average recoveries were greater than 75 % and 77 % (at LOQ and 10*LOQ μg/kg respectively) for most compounds, with acceptable values within the 70–120 % range and with an associated precision of RSD <20 % (with values from 2.8 to 15 %) (Table S2). The reproducibility of the LC-MS/MS method was evaluated by measuring the RSDwR between runs over 5 days and was <20 %, with most values < 15 %. Repeatability (RSDr) determined by performing five runs over 1 day was <14 %. Linearity was checked in the range 1–100 μg/kg for pesticides, and 0.25–100 μg/kg for mycotoxins (except deoxynivalenol, whose linearity range was 50–10,000 μg/kg). Good linearity was achieved in all cases with correlation coefficients greater than 0.988 and residuals lower than 20 % in the range studied. The matrix effect (ME) was also evaluated and, thanks to the 5-folds dilution prior to the analysis and the injection of a small volume of sample for the analysis (2.5 μL), it could be considered “soft” (<20 %), so matrix-matched calibration graphs could be used for an accurate identification and quantification of the target compounds.

**Fig. 5:**
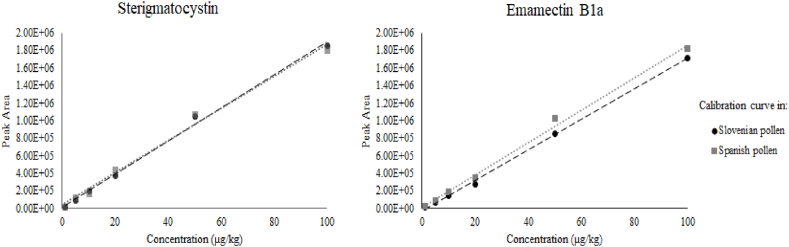
Comparison of matrix-matched calibration curves prepared with different bee pollen extracts (example reporting one mycotoxin and one pesticide included in the method).

The comparison between matrix-matched calibration curves prepared using extracts from different bee pollen samples (one Slovenian pollen and one Spanish pollen with different moisture contents) also showed good results in terms of curves overlap for the great majority of the analytes (Fig. 5). Hence, calibration curves prepared in a single matrix could be used for the quantification of the compounds of the interest in all the analyzed samples.

Reliable compounds identification was ensured by the simultaneous fulfilment of the conditions described in paragraph 2.5: co-elution of 2 precursor-ion/product-ion transitions, perfect peak overlap, same peak shape, signal-to-noise ratio greater than 3, retention time tolerance of ±0.1 min compared with the standard, and, more importantly, ion ratio of the sample extracts within ±30 % (relative) of the average of the calibration standards.

The expanded measurement uncertainty, calculated for all analytes included in the method, remained in the range 11–50 %, not exceeding the 50 % default value reported in Document SANTE/11312/2021 V2. The expanded measurement uncertainty calculated for groups of compounds with the same LOQ (mycotoxins with LOQ = 1 μg/kg, mycotoxins with LOQ = 100 μg/kg, pesticides with LOQ = 5 μg/kg), stayed in the range 28–36 % at LOQ, and 20–30 % at 10*LOQ, showing that a low uncertainty was associated to the optimized method, overall (Table S3).

### 3.5 Real samples analysis

The final validated LC-MS/MS method, including 291 compounds (282 pesticides and 9 mycotoxins), was used to analyze 34 bee pollen samples collected from Slovenia (20) and Spain (14).

The analyses of real samples revealed the presence of 41 pesticides of various classes - fungicides (39.0 %), herbicides (26.8 %), insecticides (21.9 %), and acaricides (12.2 %) - with concentrations ranging from 5.1 μg/kg to 271.98 μg/kg (Table S5). Of them, the vast majority (87.8 %) was represented by compounds that are usually employed as pesticides in agriculture, while 12.2 % of the detected chemicals, comprehending tau-fluvalinate, amitraz (DMF and DMPF), acrinathrin, and coumaphos, are used as veterinary treatments in beekeeping. One mycotoxin, ochratoxin A, was detected in 23.6 % of the analyzed samples, being the most detected contaminant in the bee pollens included in this study (concentration range: 0.99 μg/kg - 3.22 μg/kg).

Regarding the distribution of the detected compounds in the analyzed samples, three of the analyzed bee pollens contained no detectable concentration of pesticides and mycotoxins (none of the two transitions established for the target compounds were observed at the defined retention time), while 91.2 % were contaminated with at least one of the target compounds. Particularly, an average of 2.3 contaminants were found in the analyzed samples, with only one bee pollen (SP-14) containing more than 6 different compounds.

**Fig. 6:**
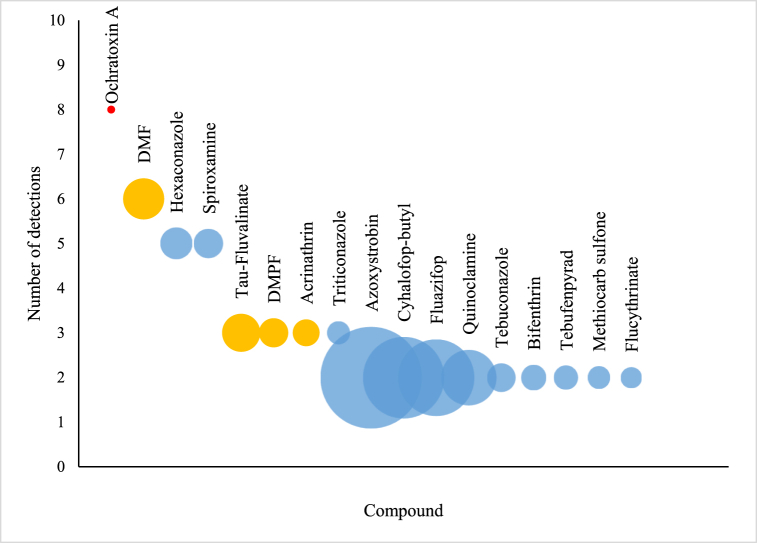
Number of positives (y-axis) and median concentration (size of the bubble) for pesticides with a number of detections >1 (Yellow was used for veterinary treatments, red for mycotoxins, and blue for plant protection chemicals).

A general overview of the results is presented in Fig. 6, where the number of detections (y-axis) and the relative median concentration (size of the bubble), is reported for each detected compound (with N of detections >1).

Typical veterinary treatments used in beekeeping to mitigate the effect of *Varroa* and other infestations were detected in 35.3 % of bee pollen samples. Particularly, DMF presented the highest occurrence in the analyzed samples, followed by tau-fluvalinate, DMPF, acrinathrin and coumaphos. Interestingly, while 64.3 % of Spanish bee pollens presented residual contamination with such compounds, only 10 % of Slovenian samples contained detectable concentrations of veterinary treatments. This is possibly due to the different climatic conditions existing in these countries, which can influence *Varroa* proliferation, and reduce or increase the need of veterinary products in beekeeping. Several studies proved that *Varroa* is sensitive to both temperature and humidity changes, but many and diverse factors can concur to its fall [34].

Due to the widespread use of pesticides in agricultural fields and residential areas, and to the long distances covered by honey bees during foraging, several plant protection chemicals were detected in bee pollen samples. As shown in Fig. 6, hexaconazole and spiroxamine were the most detected pesticides in the collected samples, while azoxystrobin was the pesticide detected at the highest median concentration. All these compounds present fungicide activity. Among the detected plant protection chemicals, a number of compounds that have been officially banned in Europe (e.g. hexaconazole, fluazifop, metolachlor etc.) could be found, which could be a combined effect of agriculturists’ misconduct, compound persistence in the environment, and direct application after the enactment of an emergency authorization. On the other hand, all the analyzed bee pollen samples resulted free from banned neonicotinoids (e.g. thiamethoxam and imidacloprid), a group of pesticides that can affect honey bee colonies and wild pollinators [35]. Only one bee pollen samples showed the presence of two neonicotinoids (acetamiprid and thiacloprid), which were both detected at very low concentrations (<10 μg/kg).

Among the mycotoxins included in the validated analytical method, only ochratoxin A could be detected in the analyzed bee pollen samples. This mycotoxin, produced by *Aspergillus* and *Penicillium* spp., occurs in a variety of food products and it is known for its nephrotoxicity and carcinogenicity [36]. Maximum levels of ochratoxin A are set by the EU for a number of food items (from 0.5 to 80 μg/kg depending on the foodstuff), but bee pollen is not included among them [28]. Both fresh and dry pollen samples analyzed in this study presented generally low levels of ochratoxin A, with a median concentration of 1.32 μg/kg. However, due to its carcinogenic nature, no level of exposure can be considered safe.

## 4 Conclusions

In this study, the first LC-MS/MS method for the simultaneous detection and quantification of a wide range of pesticides and mycotoxins in bee pollen is presented.

The optimization of such method went through several steps whose objective was finding the best experimental conditions to effectively extract and analyze the target contaminants from a complex matrix like bee pollen. Particularly, thanks to its full validation, the optimized LC-MS/MS method proved to be reliable in identifying and quantifying both pesticides and mycotoxins up to very low concentrations (LOQs = 1–5 μg/kg), in the beekeeping product under analysis.

Once validated, the optimized LC-MS/MS method was employed for the analysis of 34 bee pollen samples. The results collectively showed the presence of 41 pesticides, including a variety of veterinary treatments employed in beekeeping and plant protection chemicals, and 1 mycotoxin – ochratoxin A - in the analyzed bee pollen samples.

Thanks to their sensitivity and rapidity, simultaneous LC-MS/MS methods for the analysis of different classes of contaminants in food products, appear to be promising tools for the quality control of primary processed foods, like bee pollen, and for the safeguard of consumers’ safety.

## Disclaimer

Sample collection and data analysis were performed following the principles of statistical ethics. Samples were chosen in order to obtain the greatest amount and variety of samples possible, based on the availability of bee pollen in herbal stores, grocery stores, and online stores. The authors had absolutely no intention of making a selection that could undermine specific countries or companies.

## Data availability statement

No data was used for the research described in the article.

**Ethics declaration:** Review and/or approval by an ethics committee was not needed for this study because it did not include animal or human participation.

## CRediT authorship contribution statement

**Maria Antonietta Carrera:** Writing – original draft, Validation, Methodology, Investigation, Formal analysis. **José Antonio Martinez Martinez:** Methodology, Investigation. **María Dolores Hernando:** Writing – review & editing, Supervision, Resources, Project administration, Methodology, Funding acquisition, Conceptualization. **Amadeo R. Fernández-Alba:** Writing – review & editing, Validation, Supervision, Resources, Methodology, Conceptualization.

## Declaration of competing interest

The authors declare that they have no known competing financial interests or personal relationships that could have appeared to influence the work reported in this paper.
