# Supplementary material for: Simultaneous analysis of pesticides and mycotoxins in primary processed foods: The case of bee pollen
Source: Heliyon. 2024 Jun 26;10(13):e33512. doi: 10.1016/j.heliyon.2024.e33512 (PMC11260969; doi:10.1016/j.heliyon.2024.e33512)
Supplement: Multimedia component 1 [file mmc1.docx]

*Supplementary material*

**Simultaneous analysis of pesticides and mycotoxins in primary processed foods: the case of bee pollen**

**Maria Antonietta CARRERA^a^, José Antonio MARTINEZ MARTINEZ^b^, María Dolores HERNANDO^a^, Amadeo R. FERNÁNDEZ-ALBA^b*^**

^a^ Department of Desertification and Geo-ecology, Experimental Station of Arid Zones, CSIC, Ctra. Sacramento s/n, La Cañada de San Urbano, 04120, Almería, Spain.

^b^ European Union Reference Laboratory for Pesticide Residues in Fruit & Vegetables Agrifood Campus of International Excellence (ceiA3), Department of Chemistry and Physics, University of Almeria, Ctra. Sacramento s/n, La Cañada de San Urbano, 04120, Almería, Spain

* Corresponding author: amadeo@ual.es

Table S 1: Bee pollen samples’ details

| **ID** | **Country** | **Region** | **Dehydrated** | **Moisture content (%)** |
| --- | --- | --- | --- | --- |
| SL-01 | Slovenia | Savinjska | No | 18.9 |
| SL-02 | Slovenia | Pomurska | No | 18.9 |
| SL-03 | Slovenia | Savinjska | No | 8.17 |
| SL-04 | Slovenia | Osrednjeslovenska | No | 14.6 |
| SL-05 | Slovenia | Pomurska | No | 19.1 |
| SL-06 | Slovenia | Savinjska | No | 23.5 |
| SL-07 | Slovenia | Osrednjeslovenska | No | 18.3 |
| SL-08 | Slovenia | Jugovzhodna | No | 13.5 |
| SL-09 | Slovenia | Osrednjeslovenska | No | 19.2 |
| SL-10 | Slovenia | Savinjska | No | 18.8 |
| SL-11 | Slovenia | Osrednjeslovenska | No | 15.1 |
| SL-12 | Slovenia | Zasavska | No | 8.18 |
| SL-13 | Slovenia | Osrednjeslovenska | No | 17.1 |
| SL-14 | Slovenia | Osrednjeslovenska | No | 10.8 |
| SL-15 | Slovenia | Obalno-kraska | No | 12.4 |
| SL-16 | Slovenia | Obalno-kraska | No | 19.1 |
| SL-17 | Slovenia | Savinjska | No | 20.4 |
| SL-18 | Slovenia | Pomurska | No | 15.1 |
| SL-19 | Slovenia | Savinjska | No | 13.9 |
| SL-20 | Slovenia | Obalno-kraska | No | 15.6 |
| SP-01 | Spain | NS^a^ | Yes | 0.82 |
| SP-02 | Spain | NS | Yes | 1.9 |
| SP-03 | Spain | NS | Yes | 3.79 |
| SP-04 | Spain | NS | Yes | 1.57 |
| SP-05 | Spain | NS | Yes | 1.71 |
| SP-06 | Spain | NS | Yes | 1.25 |
| SP-07 | Spain | NS | Yes | 3.32 |
| SP-08 | Spain | NS | Yes | 1.75 |
| SP-09 | Spain | NS | Yes | 2.02 |
| SP-10 | Spain | NS | Yes | 3.62 |
| SP-11 | Spain | NS | Yes | 2.36 |
| SP-12 | Spain | NS | Yes | 2.84 |
| SP-13 | Spain | Castilla y León | Yes | 3.29 |
| SP-14 | Spain | NS | Yes | 5.82 |

*^a^NS: Not Specified*

Table S 2: Analytical and validation parameters for the compounds included in the LC-MS/MS method

| **Compound** | **Polarity** | **RT (min)** | **Precursor ion (m/z)** | **Fragment ion (m/z)** | **Dwell time (ms)** | **DP (V)** | **EP (V)** | **CE (V)** | **CXP (V)** | **LOQ (µg/kg)** | **R^2^** | **RSDwR (%)** | **RSDr (%)** | **Recovery at LOQ (%)** | **U’ at LOQ (%)** |
| --- | --- | --- | --- | --- | --- | --- | --- | --- | --- | --- | --- | --- | --- | --- | --- |
| 2,4-D | Neg | 5.94 | 221.0 | 163.0 | 6.40 | -5 | -10 | -20 | -19 | 5 | 0.989 | 2.7 | 3.2 | 75 | 50.3 |
|  |  |  |  | 160.8 | 6.40 | -5 | -10 | -20 | -19 |  |  |  |  |  |  |
| 8-Quinolinol | Pos | 5.63 | 146.0 | 127.9 | 5.79 | 1 | 10 | 33 | 14 | 5 | 0.989 | 11.3 | 9.3 | 79 | 47.7 |
|  |  |  |  | 118.0 | 5.79 | 1 | 10 | 31 | 14 |  |  |  |  |  |  |
| Acephate | Pos | 3.07 | 183.9 | 143.0 | 28.37 | 1 | 10 | 13 | 8 | 5 | 0.998 | 14.5 | 13.9 | 91 | 34.1 |
|  |  |  |  | 95.0 | 24.39 | 1 | 10 | 33 | 10 |  |  |  |  |  |  |
| Acetamiprid | Pos | 4.33 | 223.0 | 125.9 | 11.07 | 46 | 10 | 27 | 12 | 5 | 0.998 | 13.1 | 11.0 | 87 | 36.9 |
|  |  |  |  | 90.0 | 11.07 | 46 | 10 | 49 | 10 |  |  |  |  |  |  |
| Acrinathrin | Pos | 13.53 | 558.9 | 208.0 | 24.83 | 136 | 10 | 21 | 20 | 5 | 0.989 | 10.5 | 8.5 | 111 | 30.4 |
|  |  |  |  | 180.9 | 24.83 | 136 | 10 | 51 | 20 |  |  |  |  |  |  |
| Aflatoxin B1 | Pos | 5.29 | 313.0 | 285.0 | 6.92 | 111 | 10 | 33 | 28 | 1 | 0.997 | 13.2 | 13.0 | 103 | 27.1 |
|  |  |  |  | 241.0 | 6.92 | 111 | 10 | 51 | 28 |  |  |  |  |  |  |
| Alachlor | Pos | 8.88 | 270.0 | 238.1 | 3.00 | 26 | 10 | 17 | 22 | 5 | 0.989 | 11.3 | 9.5 | 84 | 39.2 |
|  |  |  |  | 162.1 | 3.00 | 26 | 10 | 29 | 18 |  |  |  |  |  |  |
| Albendazole | Pos | 6.92 | 266.0 | 234.1 | 6.88 | 76 | 10 | 27 | 24 | 5 | 0.996 | 7.6 | 7.2 | 83 | 37.2 |
|  |  |  |  | 191.0 | 6.86 | 76 | 10 | 49 | 22 |  |  |  |  |  |  |
| Aldicarb | Pos | 4.81 | 116.0 | 89.1 | 13.30 | 36 | 10 | 13 | 10 | 5 | 0.997 | 10.3 | 11.2 | 97 | 21.5 |
|  |  |  |  | 70.1 | 13.30 | 36 | 10 | 13 | 8 |  |  |  |  |  |  |
| Ametoctradin | Pos | 10.97 | 276.1 | 176.1 | 7.22 | 151 | 10 | 51 | 16 | 5 | 0.997 | 14.2 | 12.8 | 85 | 41.3 |
|  |  |  |  | 149.0 | 7.22 | 151 | 10 | 49 | 16 |  |  |  |  |  |  |
| Amisulbrom | Pos | 10.98 | 467.8 | 228.9 | 7.25 | 51 | 10 | 25 | 24 | 5 | 0.989 | 11.4 | 10.9 | 107 | 26.8 |
|  |  |  |  | 148.0 | 7.22 | 51 | 10 | 67 | 14 |  |  |  |  |  |  |
| Anilofos | Pos | 9.74 | 367.9 | 199.1 | 3.66 | 31 | 10 | 19 | 26 | 5 | 0.996 | 13.6 | 12.7 | 107 | 30.6 |
|  |  |  |  | 124.9 | 3.66 | 31 | 10 | 45 | 14 |  |  |  |  |  |  |
| Apicidin | Pos | 10.31 | 624.2 | 84.1 | 4.43 | 161 | 10 | 103 | 10 | 5 | 0.989 | 14.5 | 13.8 | 105 | 30.7 |
|  |  |  |  | 211.1 | 4.43 | 161 | 10 | 51 | 22 |  |  |  |  |  |  |
| Atrazine | Pos | 6.33 | 216.0 | 174.1 | 7.29 | 71 | 10 | 25 | 18 | 5 | 0.998 | 14.8 | 13.9 | 86 | 40.7 |
|  |  |  |  | 104.0 | 7.29 | 71 | 10 | 39 | 12 |  |  |  |  |  |  |
| Avermectin_b1a | Pos | 14.11 | 890.3 | 305.3 | 29.36 | 41 | 10 | 35 | 28 | 5 | 0.993 | 8.9 | 8.8 | 101 | 17.9 |
|  |  |  |  | 307.3 | 29.36 | 41 | 10 | 29 | 28 |  |  |  |  |  |  |
| Azinphos_methyl | Pos | 6.98 | 317.8 | 132.1 | 6.72 | 1 | 10 | 23 | 14 | 5 | 0.996 | 14.1 | 13.2 | 79 | 50.6 |
|  |  |  |  | 77.1 | 6.70 | 1 | 10 | 51 | 10 |  |  |  |  |  |  |
| Azinphos-ethyl | Pos | 8.65 | 345.9 | 132.1 | 3.00 | 86 | 10 | 25 | 14 | 5 | 0.989 | 11.0 | 10.8 | 79 | 47.4 |
|  |  |  |  | 159.9 | 3.00 | 86 | 10 | 13 | 36 |  |  |  |  |  |  |
| Azoxystrobin | Pos | 7.55 | 404.0 | 372.1 | 5.29 | 1 | 10 | 21 | 28 | 5 | 0.998 | 11.3 | 11.2 | 98 | 23.0 |
|  |  |  |  | 344.0 | 5.30 | 1 | 10 | 35 | 36 |  |  |  |  |  |  |
| BAC 10 | Pos | 8.09 | 276.0 | 91.0 | 3.34 | 1 | 10 | 53 | 10 | 5 | 0.996 | 14.2 | 13.2 | 83 | 44.3 |
|  |  |  |  | 184.2 | 3.34 | 1 | 10 | 27 | 16 |  |  |  |  |  |  |
| BAC 8 | Pos | 6.02 | 248.1 | 91.0 | 6.60 | 1 | 10 | 43 | 10 | 5 | 0.998 | 10.5 | 10.1 | 78 | 48.8 |
|  |  |  |  | 156.2 | 6.60 | 1 | 10 | 27 | 14 |  |  |  |  |  |  |
| Benalaxyl | Pos | 9.96 | 326.0 | 148.1 | 3.19 | 1 | 10 | 29 | 18 | 5 | 0.996 | 12.6 | 11.8 | 100 | 25.2 |
|  |  |  |  | 294.0 | 3.19 | 1 | 10 | 15 | 36 |  |  |  |  |  |  |
| Bendiocarb | Pos | 5.30 | 224.0 | 167.1 | 6.85 | 1 | 10 | 13 | 14 | 5 | 0.999 | 8.4 | 8.6 | 88 | 29.3 |
|  |  |  |  | 108.9 | 6.85 | 1 | 10 | 23 | 16 |  |  |  |  |  |  |
| Benzovindiflupyr | Pos | 9.69 | 398.0 | 342.0 | 3.74 | 76 | 10 | 25 | 42 | 5 | 0.996 | 10.3 | 10.2 | 96 | 22.1 |
|  |  |  |  | 321.9 | 3.74 | 76 | 10 | 33 | 30 |  |  |  |  |  |  |
| Bifenazate | Pos | 8.40 | 301.0 | 198.0 | 3.00 | 91 | 10 | 15 | 15 | 5 | 0.997 | 13.7 | 13.9 | 93 | 30.8 |
|  |  |  |  | 170.0 | 3.00 | 91 | 10 | 29 | 29 |  |  |  |  |  |  |
| Bifenazate diazene | Pos | 10.73 | 299.1 | 213.0 | 6.68 | 21 | 10 | 15 | 22 | 5 | 0.994 | 10.7 | 9.8 | 79 | 47.1 |
|  |  |  |  | 196.0 | 6.68 | 21 | 10 | 45 | 24 |  |  |  |  |  |  |
| Bifenthrin | Pos | 14.49 | 440.1 | 181.1 | 67.29 | 56 | 10 | 17 | 30 | 5 | 0.989 | 2.2 | 2.6 | 111 | 22.4 |
|  |  |  |  | 165.1 | 66.19 | 56 | 10 | 103 | 18 |  |  |  |  |  |  |
| Bitertanol | Pos | 10.27 | 338.2 | 269.1 | 4.19 | 31 | 10 | 15 | 26 | 5 | 0.989 | 11.3 | 9.7 | 108 | 27.7 |
|  |  |  |  | 99.0 | 4.19 | 31 | 10 | 21 | 12 |  |  |  |  |  |  |
| Bixafen | Pos | 9.25 | 413.8 | 393.9 | 3.64 | 1 | 10 | 21 | 38 | 5 | 0.995 | 13.2 | 11.6 | 87 | 37.1 |
|  |  |  |  | 265.9 | 3.64 | 1 | 10 | 33 | 24 |  |  |  |  |  |  |
| Boscalid | Pos | 7.76 | 342.9 | 271.0 | 4.60 | 101 | 10 | 35 | 34 | 5 | 0.992 | 11.2 | 11.1 | 92 | 27.5 |
|  |  |  |  | 307.0 | 4.60 | 86 | 10 | 29 | 28 |  |  |  |  |  |  |
| Bromacil | Pos | 5.37 | 260.9 | 205.0 | 6.53 | 31 | 10 | 19 | 20 | 5 | 0.989 | 14.8 | 13.2 | 83 | 45.1 |
|  |  |  |  | 187.8 | 6.53 | 31 | 10 | 41 | 18 |  |  |  |  |  |  |
| Bromuconazole | Pos | 8.50 | 377.9 | 159.1 | 3.00 | 1 | 10 | 37 | 12 | 5 | 0.989 | 6.4 | 7.8 | 107 | 19.0 |
|  |  |  |  | 160.9 | 3.00 | 1 | 10 | 37 | 18 |  |  |  |  |  |  |
| Bupirimate | Pos | 8.81 | 317.0 | 166.2 | 3.00 | 81 | 10 | 33 | 10 | 5 | 0.995 | 12.5 | 12.1 | 95 | 26.9 |
|  |  |  |  | 210.1 | 3.00 | 81 | 10 | 33 | 26 |  |  |  |  |  |  |
| Buprofezin | Pos | 11.62 | 306.0 | 201.1 | 8.58 | 36 | 10 | 17 | 12 | 5 | 0.999 | 8.8 | 8.9 | 88 | 29.8 |
|  |  |  |  | 116.0 | 8.58 | 36 | 10 | 23 | 8 |  |  |  |  |  |  |
| Butoxycarboxim | Pos | 3.42 | 223.0 | 64.9 | 8.39 | 26 | 10 | 23 | 8 | 5 | 0.991 | 7.9 | 8.4 | 99 | 15.9 |
|  |  |  |  | 106.0 | 8.16 | 26 | 10 | 13 | 12 |  |  |  |  |  |  |
| Carbaryl | Pos | 5.62 | 202.0 | 145.0 | 5.80 | 1 | 10 | 17 | 16 | 5 | 0.998 | 12.3 | 11.6 | 83 | 42.0 |
|  |  |  |  | 127.0 | 5.80 | 1 | 10 | 41 | 14 |  |  |  |  |  |  |
| Carbendazim | Pos | 3.79 | 192.0 | 160.1 | 7.42 | 41 | 10 | 25 | 16 | 5 | 0.996 | 11.0 | 11.2 | 79 | 47.4 |
|  |  |  |  | 132.1 | 7.42 | 41 | 10 | 41 | 16 |  |  |  |  |  |  |
| carbendazim d3 | Pos | 3.78 | 194.8 | 159.9 | 7.36 | 1 | 10 | 23 | 30 | - | - | - | - | - | - |
|  |  |  |  | 132.0 | 7.36 | 1 | 10 | 43 | 14 |  |  |  |  |  |  |
| Chlorantraniliprole | Pos | 6.97 | 481.9 | 450.9 | 6.73 | 56 | 10 | 29 | 32 | 5 | 0.999 | 8.7 | 9.4 | 98 | 17.9 |
|  |  |  |  | 283.9 | 6.73 | 56 | 10 | 19 | 24 |  |  |  |  |  |  |
| Chlorbromuron | Pos | 7.52 | 294.8 | 205.9 | 5.36 | 41 | 10 | 27 | 10 | 5 | 0.998 | 4.6 | 5.1 | 84 | 33.3 |
|  |  |  |  | 182.1 | 5.36 | 41 | 10 | 25 | 10 |  |  |  |  |  |  |
| Chlorfenvinphos | Pos | 9.95 | 358.8 | 155.1 | 3.22 | 56 | 10 | 17 | 14 | 5 | 0.989 | 13.8 | 12.4 | 110 | 34.1 |
|  |  |  |  | 170.0 | 3.23 | 56 | 10 | 59 | 16 |  |  |  |  |  |  |
| Chlorfluazuron | Pos | 12.97 | 541.8 | 384.8 | 22.94 | 1 | 10 | 31 | 36 | 5 | 0.989 | 12.6 | 11.7 | 111 | 33.5 |
|  |  |  |  | 158.0 | 22.94 | 1 | 10 | 27 | 16 |  |  |  |  |  |  |
| Chloridazon | Pos | 4.33 | 222.0 | 104.0 | 11.07 | 81 | 10 | 31 | 10 | 5 | 0.998 | 11.9 | 11.4 | 80 | 46.5 |
|  |  |  |  | 91.9 | 11.07 | 81 | 10 | 33 | 10 |  |  |  |  |  |  |
| Chlorotoluron | Pos | 6.04 | 213.0 | 72.0 | 6.64 | 61 | 10 | 23 | 8 | 5 | 0.997 | 10.3 | 10.7 | 83 | 39.8 |
|  |  |  |  | 139.9 | 6.64 | 61 | 10 | 35 | 12 |  |  |  |  |  |  |
| Chloroxuron | Pos | 8.25 | 290.9 | 72.1 | 3.00 | 1 | 10 | 47 | 8 | 5 | 0.995 | 7.9 | 8.2 | 88 | 28.7 |
|  |  |  |  | 164.2 | 3.00 | 1 | 10 | 23 | 14 |  |  |  |  |  |  |
| Chlorpyriphos methyl | Pos | 10.41 | 321.8 | 125.0 | 5.08 | 1 | 10 | 29 | 12 | 5 | 0.989 | 8.6 | 7.4 | 93 | 22.2 |
|  |  |  |  | 289.9 | 5.08 | 1 | 10 | 21 | 10 |  |  |  |  |  |  |
| Chlorpyriphos | Pos | 12.01 | 351.8 | 199.8 | 10.59 | 51 | 10 | 29 | 12 | 5 | 0.993 | 13.4 | 13.1 | 106 | 29.4 |
|  |  |  |  | 96.9 | 10.70 | 51 | 10 | 47 | 10 |  |  |  |  |  |  |
| Chromafenozide | Pos | 8.75 | 395.1 | 91.1 | 3.00 | 21 | 10 | 87 | 12 | 5 | 0.996 | 2.8 | 4.1 | 105 | 11.5 |
|  |  |  |  | 175.0 | 3.00 | 21 | 10 | 19 | 10 |  |  |  |  |  |  |
| Clofentezine | Pos | 10.12 | 303.0 | 138.1 | 3.45 | 76 | 10 | 21 | 16 | 5 | 0.989 | 11.0 | 9.7 | 88 | 32.6 |
|  |  |  |  | 102.0 | 3.45 | 76 | 10 | 51 | 12 |  |  |  |  |  |  |
| Clomazone | Pos | 7.13 | 240.0 | 124.9 | 6.96 | 61 | 10 | 27 | 14 | 5 | 0.999 | 6.3 | 7.1 | 87 | 28.9 |
|  |  |  |  | 89.1 | 6.96 | 61 | 10 | 65 | 10 |  |  |  |  |  |  |
| Coumaphos | Pos | 10.01 | 362.9 | 226.9 | 3.15 | 121 | 10 | 35 | 20 | 5 | 0.990 | 4.9 | 4.7 | 91 | 20.5 |
|  |  |  |  | 306.9 | 3.15 | 121 | 10 | 25 | 18 |  |  |  |  |  |  |
| Cyantraniliprole | Pos | 5.77 | 474.8 | 285.9 | 5.82 | 1 | 10 | 21 | 28 | 5 | 0.996 | 12.0 | 10.8 | 107 | 27.8 |
|  |  |  |  | 443.9 | 5.82 | 1 | 10 | 27 | 44 |  |  |  |  |  |  |
| Cyazofamid | Pos | 8.82 | 324.9 | 108.0 | 3.00 | 26 | 10 | 19 | 12 | 5 | 0.989 | 3.7 | 4.9 | 115 | 30.9 |
|  |  |  |  | 261.0 | 3.00 | 26 | 10 | 13 | 32 |  |  |  |  |  |  |
| Cyflufenamid | Pos | 10.26 | 413.7 | 296.0 | 4.13 | 1 | 10 | 21 | 26 | 5 | 0.989 | 10.8 | 8.9 | 87 | 33.8 |
|  |  |  |  | 241.0 | 4.13 | 1 | 10 | 31 | 22 |  |  |  |  |  |  |
| Cyflumetofen | Pos | 11.37 | 464.7 | 172.9 | 8.83 | 1 | 10 | 29 | 20 | 5 | 0.989 | 5.9 | 6.2 | 112 | 26.7 |
|  |  |  |  | 249.0 | 8.83 | 1 | 10 | 19 | 22 |  |  |  |  |  |  |
| Cyhalofop-butyl | Pos | 11.05 | 375.1 | 256.0 | 7.29 | 31 | 10 | 25 | 25 | 5 | 0.989 | 12.1 | 12.4 | 114 | 37.0 |
|  |  |  |  | 358.0 | 7.29 | 31 | 10 | 11 | 11 |  |  |  |  |  |  |
| Cymoxanil | Pos | 4.42 | 199.0 | 128.0 | 12.82 | 26 | 10 | 13 | 14 | 5 | 0.999 | 10.8 | 9.6 | 91 | 28.1 |
|  |  |  |  | 111.0 | 12.82 | 26 | 10 | 25 | 14 |  |  |  |  |  |  |
| Cypermethrin | Pos | 13.26 | 433.0 | 190.9 | 25.35 | 26 | 10 | 21 | 10 | 5 | 0.989 | 5.9 | 6.0 | 94 | 16.8 |
|  |  |  |  | 416.0 | 25.15 | 26 | 10 | 13 | 16 |  |  |  |  |  |  |
| Cyproconazole | Pos | 8.29 | 291.9 | 70.1 | 3.00 | 1 | 10 | 45 | 10 | 5 | 0.990 | 8.4 | 7.2 | 104 | 18.6 |
|  |  |  |  | 125.0 | 3.00 | 1 | 10 | 43 | 14 |  |  |  |  |  |  |
| Cyprodinil | Pos | 9.05 | 226.0 | 93.0 | 3.30 | 96 | 10 | 53 | 10 | 5 | 0.995 | 10.4 | 9.4 | 78 | 48.7 |
|  |  |  |  | 108.1 | 3.30 | 96 | 10 | 35 | 12 |  |  |  |  |  |  |
| Dazomet | Pos | 3.56 | 163.0 | 90.0 | 7.33 | 31 | 10 | 10 | 25 | 5 | 0.989 | 9.6 | 9.2 | 78 | 48.0 |
|  |  |  |  | 120.0 | 7.32 | 31 | 10 | 10 | 27 |  |  |  |  |  |  |
| DEET | Pos | 6.59 | 192.0 | 119.1 | 6.59 | 1 | 10 | 25 | 14 | 5 | 0.997 | 14.3 | 13.2 | 87 | 38.7 |
|  |  |  |  | 91.1 | 6.59 | 1 | 10 | 39 | 10 |  |  |  |  |  |  |
| Demeton-S-methyl | Pos | 5.52 | 230.9 | 88.9 | 6.14 | 1 | 10 | 23 | 10 | 5 | 0.997 | 12.4 | 11.8 | 91 | 30.6 |
|  |  |  |  | 61.0 | 6.14 | 1 | 10 | 43 | 8 |  |  |  |  |  |  |
| Demeton-S-methylsulfone | Pos | 3.70 | 262.9 | 168.9 | 7.09 | 36 | 10 | 21 | 20 | 5 | 0.997 | 9.7 | 8.7 | 102 | 19.8 |
|  |  |  |  | 108.9 | 7.07 | 36 | 10 | 37 | 12 |  |  |  |  |  |  |
| Demeton-S-methylsulfoxide | Pos | 3.64 | 246.9 | 169.0 | 7.12 | 11 | 10 | 19 | 16 | 5 | 0.998 | 10.2 | 10.4 | 95 | 22.7 |
|  |  |  |  | 109.1 | 7.12 | 11 | 10 | 37 | 12 |  |  |  |  |  |  |
| Deoxynivalenol | Pos | 3.51 | 297.0 | 203.0 | 7.46 | 65 | 10 | 22 | 11 | 100 | 0.998 | 11.5 | 9.8 | 96 | 24.4 |
|  |  |  |  | 231.0 | 7.46 | 65 | 10 | 18 | 21 |  |  |  |  |  |  |
| Desethylterbuthylazine | Pos | 5.63 | 202.0 | 146.0 | 5.79 | 26 | 10 | 23 | 14 | 5 | 0.996 | 7.9 | 7.8 | 80 | 43.0 |
|  |  |  |  | 104.0 | 6.51 | 26 | 10 | 37 | 12 |  |  |  |  |  |  |
| Diazinon | Pos | 9.91 | 304.9 | 169.0 | 3.33 | 1 | 10 | 29 | 16 | 5 | 0.997 | 14.9 | 13.5 | 99 | 29.9 |
|  |  |  |  | 153.1 | 3.33 | 1 | 10 | 29 | 14 |  |  |  |  |  |  |
| Dichlorvos | Pos | 5.29 | 220.8 | 109.1 | 6.92 | 26 | 10 | 25 | 12 | 5 | 0.989 | 11.3 | 10.9 | 113 | 34.4 |
|  |  |  |  | 79.1 | 6.92 | 26 | 10 | 37 | 10 |  |  |  |  |  |  |
| Dichlorvos d6 | Pos | 5.24 | 226.9 | 115.1 | 7.53 | 41 | 10 | 25 | 12 | - | - | - | - | - | - |
|  |  |  |  | 82.9 | 7.62 | 41 | 10 | 39 | 14 |  |  |  |  |  |  |
| Dicrotophos | Pos | 3.95 | 238.0 | 112.0 | 8.23 | 1 | 10 | 17 | 12 | 5 | 0.997 | 9.7 | 10.2 | 93 | 23.9 |
|  |  |  |  | 193.1 | 8.28 | 1 | 10 | 15 | 16 |  |  |  |  |  |  |
| Diethofencarb | Pos | 7.37 | 268.0 | 226.1 | 6.24 | 26 | 10 | 15 | 20 | 5 | 0.999 | 7.6 | 8.2 | 84 | 35.4 |
|  |  |  |  | 124.0 | 6.24 | 26 | 10 | 43 | 14 |  |  |  |  |  |  |
| Difenoconazole | Pos | 10.76 | 405.9 | 251.1 | 6.77 | 96 | 10 | 35 | 22 | 5 | 0.995 | 11.0 | 10.8 | 98 | 22.4 |
|  |  |  |  | 188.0 | 6.81 | 96 | 10 | 61 | 18 |  |  |  |  |  |  |
| Difenoxuron | Pos | 6.57 | 287.0 | 123.0 | 6.64 | 81 | 10 | 27 | 16 | 5 | 0.998 | 14.5 | 13.1 | 88 | 37.6 |
|  |  |  |  | 72.1 | 6.64 | 81 | 10 | 49 | 8 |  |  |  |  |  |  |
| Diflubenzuron | Neg | 8.87 | 308.9 | 156.0 | 3.00 | -30 | -10 | -16 | -19 | 5 | 0.988 | 12.8 | 10.4 | 113 | 36.5 |
|  |  |  |  | 288.9 | 3.00 | -30 | -10 | -14 | -35 |  |  |  |  |  |  |
| Dimethoate | Pos | 4.25 | 229.9 | 199.0 | 9.91 | 26 | 10 | 13 | 16 | 5 | 0.999 | 9.8 | 7.5 | 85 | 35.8 |
|  |  |  |  | 125.1 | 9.91 | 26 | 10 | 29 | 14 |  |  |  |  |  |  |
| Dimethoate-d6 | Pos | 4.26 | 236.0 | 204.9 | 10.01 | 86 | 10 | 10 | 33 | - | - | - | - | - | - |
|  |  |  |  | 130.9 | 10.01 | 86 | 10 | 10 | 23 |  |  |  |  |  |  |
| Dimethomorph | Pos | 7.91 | 388.0 | 301.0 | 3.78 | 1 | 10 | 29 | 24 | 5 | 0.995 | 13.2 | 11.9 | 91 | 32.0 |
|  |  |  |  | 165.1 | 3.81 | 1 | 10 | 43 | 16 |  |  |  |  |  |  |
| Dimethylvinphos-Z | Pos | 8.18 | 330.8 | 127.0 | 3.12 | 26 | 10 | 17 | 14 | 5 | 0.996 | 9.6 | 9.8 | 97 | 20.1 |
|  |  |  |  | 169.9 | 3.00 | 26 | 10 | 57 | 18 |  |  |  |  |  |  |
| Diniconazole | Pos | 10.41 | 325.9 | 70.1 | 5.08 | 76 | 10 | 67 | 10 | 5 | 0.989 | 6.3 | 7.5 | 98 | 13.2 |
|  |  |  |  | 159.0 | 5.08 | 76 | 10 | 43 | 16 |  |  |  |  |  |  |
| Dinotefuran | Pos | 3.34 | 203.0 | 129.1 | 9.96 | 31 | 10 | 17 | 20 | 5 | 0.989 | 11.7 | 11.4 | 114 | 36.5 |
|  |  |  |  | 113.1 | 9.96 | 31 | 10 | 17 | 12 |  |  |  |  |  |  |
| Diuron | Pos | 5.86 | 232.9 | 72.1 | 6.00 | 1 | 10 | 21 | 8 | 5 | 0.998 | 10.3 | 9.2 | 84 | 38.1 |
|  |  |  |  | 159.9 | 5.94 | 1 | 10 | 37 | 16 |  |  |  |  |  |  |
| DMF | Pos | 5.03 | 150.0 | 107.1 | 10.89 | 71 | 10 | 29 | 12 | 5 | 0.999 | 8.4 | 8.8 | 108 | 23.2 |
|  |  |  |  | 106.1 | 10.89 | 71 | 10 | 43 | 12 |  |  |  |  |  |  |
| DMPF | Pos | 3.72 | 163.0 | 121.9 | 7.15 | 1 | 10 | 23 | 22 | 5 | 0.996 | 9.7 | 10.6 | 78 | 48.1 |
|  |  |  |  | 106.1 | 7.15 | 1 | 10 | 43 | 16 |  |  |  |  |  |  |
| Edifenphos | Pos | 9.77 | 310.9 | 282.9 | 3.59 | 36 | 10 | 19 | 16 | 5 | 0.996 | 8.4 | 7.6 | 93 | 21.9 |
|  |  |  |  | 109.0 | 3.59 | 36 | 10 | 45 | 12 |  |  |  |  |  |  |
| Emamectin B1a | Pos | 12.10 | 886.4 | 158.2 | 11.78 | 111 | 10 | 41 | 16 | 5 | 0.996 | 11.9 | 10.2 | 79 | 48.3 |
|  |  |  |  | 82.2 | 11.78 | 111 | 10 | 117 | 10 |  |  |  |  |  |  |
| Emamectin B1b | Pos | 11.64 | 872.3 | 158.1 | 8.60 | 96 | 10 | 41 | 16 | 5 | 0.989 | 14.3 | 11.4 | 102 | 28.9 |
|  |  |  |  | 82.1 | 8.59 | 96 | 10 | 111 | 10 |  |  |  |  |  |  |
| Enniatin A | Pos | 13.69 | 668.2 | 210.1 | 24.54 | 231 | 10 | 33 | 26 | 1 | 0.989 | 12.4 | 13.1 | 98 | 25.1 |
|  |  |  |  | 228.1 | 24.54 | 231 | 10 | 37 | 28 |  |  |  |  |  |  |
| Enniatin B | Pos | 12.88 | 640.2 | 196.1 | 23.41 | 206 | 10 | 33 | 16 | 1 | 0.990 | 10.8 | 10.2 | 95 | 23.8 |
|  |  |  |  | 214.1 | 23.41 | 206 | 10 | 35 | 22 |  |  |  |  |  |  |
| EPN | Pos | 10.68 | 323.9 | 296.0 | 6.53 | 51 | 10 | 19 | 28 | 5 | 0.989 | 9.0 | 8.5 | 119 | 42.0 |
|  |  |  |  | 157.0 | 6.53 | 51 | 10 | 31 | 14 |  |  |  |  |  |  |
| Epoxiconazole | Pos | 8.96 | 329.9 | 121.0 | 3.04 | 56 | 10 | 27 | 14 | 5 | 0.995 | 7.5 | 7.9 | 109 | 23.4 |
|  |  |  |  | 101.0 | 3.04 | 56 | 10 | 67 | 12 |  |  |  |  |  |  |
| Ethiofencarb | Pos | 5.85 | 226.0 | 107.1 | 5.94 | 31 | 10 | 25 | 10 | 5 | 0.997 | 11.0 | 10.4 | 88 | 32.6 |
|  |  |  |  | 164.0 | 5.94 | 31 | 10 | 11 | 18 |  |  |  |  |  |  |
| Ethion | Pos | 11.92 | 385.1 | 199.0 | 9.67 | 31 | 10 | 13 | 16 | 5 | 0.989 | 14.1 | 13.8 | 103 | 28.8 |
|  |  |  |  | 142.9 | 9.67 | 31 | 10 | 37 | 14 |  |  |  |  |  |  |
| Ethiprole | Pos | 7.55 | 396.9 | 350.9 | 5.29 | 111 | 10 | 29 | 28 | 5 | 0.997 | 12.1 | 11.4 | 91 | 30.2 |
|  |  |  |  | 255.0 | 5.29 | 111 | 10 | 49 | 22 |  |  |  |  |  |  |
| Ethirimol | Pos | 4.82 | 210.1 | 140.1 | 13.23 | 1 | 10 | 29 | 16 | 5 | 0.999 | 6.9 | 8.6 | 82 | 38.6 |
|  |  |  |  | 98.0 | 13.30 | 1 | 10 | 37 | 12 |  |  |  |  |  |  |
| Ethoprophos | Pos | 8.86 | 243.0 | 173.0 | 3.00 | 56 | 10 | 21 | 10 | 5 | 0.990 | 9.3 | 7.9 | 92 | 24.5 |
|  |  |  |  | 131.0 | 3.00 | 56 | 10 | 29 | 14 |  |  |  |  |  |  |
| Etofenprox | Pos | 14.30 | 394.1 | 177.1 | 46.77 | 6 | 10 | 19 | 10 | 5 | 0.989 | 10.8 | 12.1 | 104 | 23.0 |
|  |  |  |  | 359.1 | 46.77 | 6 | 10 | 15 | 28 |  |  |  |  |  |  |
| Etoxazole | Pos | 12.65 | 359.7 | 141.1 | 22.46 | 1 | 10 | 37 | 18 | 5 | 0.989 | 4.0 | 5.4 | 116 | 33.0 |
|  |  |  |  | 304.1 | 22.46 | 1 | 10 | 25 | 26 |  |  |  |  |  |  |
| Famoxadone | Pos | 9.94 | 392.2 | 330.9 | 3.23 | 21 | 10 | 13 | 30 | 5 | 0.989 | 14.7 | 13.2 | 93 | 32.6 |
|  |  |  |  | 238.0 | 3.23 | 21 | 10 | 25 | 12 |  |  |  |  |  |  |
| Fenamidone | Pos | 7.54 | 312.0 | 236.1 | 5.30 | 36 | 10 | 21 | 20 | 5 | 0.998 | 8.4 | 7.6 | 88 | 29.3 |
|  |  |  |  | 92.0 | 5.30 | 36 | 10 | 39 | 10 |  |  |  |  |  |  |
| Fenamiphos | Pos | 9.20 | 303.9 | 217.0 | 3.52 | 61 | 10 | 31 | 12 | 5 | 0.997 | 10.6 | 12.1 | 94 | 24.4 |
|  |  |  |  | 234.0 | 3.52 | 61 | 10 | 23 | 18 |  |  |  |  |  |  |
| Fenamiphos - sulfone | Pos | 5.56 | 335.9 | 266.0 | 6.00 | 91 | 10 | 29 | 14 | 5 | 0.999 | 11.7 | 9.8 | 91 | 29.5 |
|  |  |  |  | 308.1 | 6.00 | 91 | 10 | 21 | 26 |  |  |  |  |  |  |
| Fenamiphos - sulfoxide | Pos | 5.48 | 319.9 | 233.0 | 6.27 | 91 | 10 | 31 | 18 | 5 | 0.997 | 14.2 | 13.5 | 92 | 32.6 |
|  |  |  |  | 292.0 | 6.27 | 91 | 10 | 23 | 14 |  |  |  |  |  |  |
| Fenarimol | Pos | 8.84 | 330.9 | 268.0 | 3.00 | 111 | 10 | 33 | 22 | 5 | 0.989 | 10.8 | 11.2 | 89 | 30.8 |
|  |  |  |  | 189.1 | 3.00 | 111 | 10 | 65 | 18 |  |  |  |  |  |  |
| Fenazaquin | Pos | 13.87 | 307.0 | 161.1 | 25.62 | 1 | 10 | 23 | 16 | 5 | 0.989 | 8.7 | 8.9 | 93 | 22.3 |
|  |  |  |  | 131.2 | 25.62 | 1 | 10 | 63 | 16 |  |  |  |  |  |  |
| Fenbendazole | Pos | 8.09 | 299.9 | 268.0 | 3.34 | 126 | 10 | 33 | 30 | 5 | 0.997 | 9.7 | 7.5 | 79 | 46.3 |
|  |  |  |  | 158.9 | 3.34 | 126 | 10 | 45 | 18 |  |  |  |  |  |  |
| Fenbuconazole | Pos | 8.98 | 337.0 | 125.0 | 3.07 | 66 | 10 | 37 | 14 | 5 | 0.989 | 10.4 | 11.2 | 90 | 28.9 |
|  |  |  |  | 70.1 | 3.07 | 66 | 10 | 47 | 8 |  |  |  |  |  |  |
| Fenhexamid | Pos | 8.52 | 301.9 | 97.2 | 3.00 | 96 | 10 | 31 | 12 | 5 | 0.992 | 11.7 | 10.4 | 95 | 25.4 |
|  |  |  |  | 142.9 | 3.00 | 96 | 10 | 45 | 42 |  |  |  |  |  |  |
| Fenobucarb | Pos | 7.23 | 208.0 | 95.1 | 7.04 | 31 | 10 | 21 | 12 | 5 | 0.998 | 8.7 | 9.1 | 87 | 31.3 |
|  |  |  |  | 152.1 | 7.04 | 31 | 10 | 13 | 16 |  |  |  |  |  |  |
| Fenoxycarb | Pos | 9.25 | 302.0 | 116.0 | 3.64 | 36 | 10 | 15 | 12 | 5 | 0.992 | 10.2 | 9.8 | 91 | 27.2 |
|  |  |  |  | 88.2 | 3.64 | 36 | 10 | 31 | 12 |  |  |  |  |  |  |
| Fenpicoxamid | Pos | 11.52 | 614.9 | 239.0 | 8.80 | 1 | 10 | 37 | 26 | 5 | 0.991 | 9.3 | 8.6 | 97 | 19.5 |
|  |  |  |  | 515.0 | 8.80 | 1 | 10 | 25 | 20 |  |  |  |  |  |  |
| Fenpropathrin | Pos | 12.01 | 350.0 | 97.0 | 10.59 | 36 | 10 | 47 | 10 | 5 | 0.989 | 5.2 | 6.9 | 112 | 26.2 |
|  |  |  |  | 125.1 | 10.59 | 36 | 10 | 17 | 16 |  |  |  |  |  |  |
| Fenpropidin | Pos | 6.67 | 273.9 | 147.1 | 6.71 | 76 | 10 | 39 | 18 | 5 | 0.997 | 7.4 | 8.6 | 76 | 50.2 |
|  |  |  |  | 117.1 | 6.71 | 76 | 10 | 71 | 12 |  |  |  |  |  |  |
| Fenpropimorph | Pos | 7.12 | 304.1 | 147.1 | 6.93 | 1 | 10 | 39 | 10 | 5 | 0.999 | 12.5 | 11.4 | 79 | 48.9 |
|  |  |  |  | 117.1 | 6.93 | 1 | 10 | 77 | 14 |  |  |  |  |  |  |
| Fenpyrazamine | Pos | 8.41 | 332.0 | 230.1 | 3.00 | 1 | 10 | 25 | 22 | 5 | 0.992 | 7.4 | 8.9 | 89 | 26.5 |
|  |  |  |  | 231.0 | 3.00 | 1 | 10 | 17 | 26 |  |  |  |  |  |  |
| Fenpyroximate-E | Pos | 13.21 | 422.0 | 366.1 | 24.88 | 26 | 10 | 25 | 38 | 5 | 0.989 | 12.9 | 13.1 | 114 | 38.1 |
|  |  |  |  | 138.0 | 24.88 | 26 | 10 | 41 | 18 |  |  |  |  |  |  |
| Fensulfothion | Pos | 6.64 | 308.9 | 280.9 | 6.56 | 1 | 10 | 21 | 28 | 5 | 0.997 | 11.2 | 10.7 | 89 | 31.4 |
|  |  |  |  | 157.0 | 6.56 | 1 | 10 | 21 | 28 |  |  |  |  |  |  |
| Fenthion | Pos | 9.54 | 278.9 | 169.1 | 3.85 | 51 | 10 | 23 | 10 | 5 | 0.989 | 14.5 | 13.4 | 109 | 34.1 |
|  |  |  |  | 105.0 | 3.85 | 51 | 10 | 23 | 10 |  |  |  |  |  |  |
| Fenthion sulfone | Pos | 5.77 | 310.8 | 125.0 | 5.82 | 106 | 10 | 29 | 14 | 5 | 0.994 | 9.0 | 10.3 | 94 | 21.6 |
|  |  |  |  | 279.0 | 5.82 | 106 | 10 | 25 | 20 |  |  |  |  |  |  |
| Fenthion sulfoxide | Pos | 5.68 | 294.9 | 279.9 | 5.77 | 76 | 10 | 25 | 26 | 5 | 0.999 | 8.4 | 7.5 | 84 | 36.1 |
|  |  |  |  | 109.0 | 5.77 | 76 | 10 | 39 | 12 |  |  |  |  |  |  |
| Fenuron | Pos | 4.21 | 165.0 | 71.9 | 9.82 | 21 | 10 | 19 | 10 | 5 | 0.998 | 14.2 | 13.8 | 89 | 35.9 |
|  |  |  |  | 119.8 | 9.69 | 21 | 10 | 23 | 16 |  |  |  |  |  |  |
| Fipronil | Pos | 9.13 | 453.8 | 436.9 | 3.44 | 21 | 10 | 17 | 22 | 5 | 0.989 | 12.7 | 11.4 | 112 | 34.9 |
|  |  |  |  | 367.9 | 3.44 | 21 | 10 | 31 | 20 |  |  |  |  |  |  |
| Flazasulfuron | Pos | 6.89 | 407.9 | 182.0 | 6.96 | 36 | 10 | 29 | 18 | 5 | 0.998 | 10.4 | 8.6 | 78 | 48.7 |
|  |  |  |  | 139.0 | 6.96 | 36 | 10 | 61 | 16 |  |  |  |  |  |  |
| Flonicamid | Pos | 3.68 | 230.0 | 203.0 | 7.07 | 66 | 10 | 25 | 12 | 5 | 0.994 | 5.8 | 6.9 | 106 | 16.7 |
|  |  |  |  | 173.9 | 5.17 | 66 | 10 | 25 | 10 |  |  |  |  |  |  |
| Florpyrauxifen_benzyl | Pos | 10.49 | 438.8 | 91.0 | 5.47 | 56 | 10 | 63 | 12 | 5 | 0.989 | 14.2 | 13.9 | 98 | 28.7 |
|  |  |  |  | 65.0 | 5.47 | 56 | 10 | 113 | 8 |  |  |  |  |  |  |
| Fluacrypyrim | Pos | 10.74 | 427.1 | 145.1 | 6.70 | 16 | 10 | 41 | 16 | 5 | 0.993 | 9.6 | 10.5 | 104 | 20.8 |
|  |  |  |  | 204.9 | 6.74 | 16 | 10 | 15 | 12 |  |  |  |  |  |  |
| Fluazifop | Neg | 7.47 | 325.7 | 254.0 | 5.57 | -5 | -10 | -20 | -11 | 5 | 0.989 | 4.2 | 4.5 | 85 | 31.2 |
|  |  |  |  | 108.0 | 5.53 | -5 | -10 | -40 | -29 |  |  |  |  |  |  |
| Flubendiamide | Neg | 9.53 | 680.9 | 253.9 | 3.85 | -65 | -10 | -34 | -25 | 5 | 0.994 | 7.9 | 8.7 | 93 | 21.1 |
|  |  |  |  | 273.9 | 3.85 | -65 | -10 | -24 | -33 |  |  |  |  |  |  |
| Flucythrinate | Pos | 12.57 | 468.9 | 412.0 | 21.68 | 26 | 10 | 17 | 36 | 5 | 0.989 | 14.5 | 13.4 | 113 | 38.9 |
|  |  |  |  | 199.0 | 21.68 | 26 | 10 | 27 | 20 |  |  |  |  |  |  |
| Fludioxonil | Pos | 7.61 | 266.0 | 229.1 | 5.25 | 1 | 10 | 19 | 20 | 5 | 0.994 | 10.2 | 11.2 | 84 | 37.9 |
|  |  |  |  | 158.1 | 5.25 | 1 | 10 | 47 | 16 |  |  |  |  |  |  |
| Flufenacet | Pos | 8.65 | 364.7 | 153.1 | 3.00 | 1 | 10 | 33 | 26 | 5 | 0.989 | 11.8 | 10.7 | 78 | 49.9 |
|  |  |  |  | 195.0 | 3.00 | 1 | 10 | 33 | 22 |  |  |  |  |  |  |
| Flufenoxuron | Pos | 12.49 | 488.9 | 158.0 | 21.04 | 66 | 10 | 25 | 16 | 5 | 0.988 | 3.2 | 4.5 | 114 | 28.7 |
|  |  |  |  | 141.1 | 21.04 | 66 | 10 | 71 | 16 |  |  |  |  |  |  |
| Fluometuron | Pos | 5.85 | 233.0 | 72.0 | 5.94 | 56 | 10 | 23 | 8 | 5 | 0.999 | 9.3 | 8.7 | 84 | 37.0 |
|  |  |  |  | 159.9 | 5.94 | 56 | 10 | 37 | 16 |  |  |  |  |  |  |
| Fluopicolide | Pos | 8.01 | 382.8 | 173.0 | 3.53 | 26 | 10 | 31 | 18 | 5 | 0.991 | 14.2 | 13.0 | 92 | 32.6 |
|  |  |  |  | 144.9 | 3.53 | 26 | 10 | 75 | 16 |  |  |  |  |  |  |
| Fluopyram | Pos | 8.46 | 396.9 | 208.0 | 3.00 | 61 | 10 | 31 | 14 | 5 | 0.997 | 11.8 | 11.4 | 92 | 28.5 |
|  |  |  |  | 173.0 | 3.00 | 61 | 10 | 41 | 16 |  |  |  |  |  |  |
| Flupyradifurone | Pos | 4.25 | 288.9 | 125.9 | 9.91 | 1 | 10 | 27 | 14 | 5 | 0.999 | 8.6 | 8.2 | 94 | 21.0 |
|  |  |  |  | 90.0 | 9.91 | 1 | 10 | 59 | 10 |  |  |  |  |  |  |
| Fluquinconazole | Pos | 8.50 | 376.1 | 306.9 | 3.00 | 91 | 10 | 35 | 22 | 5 | 0.995 | 9.7 | 9.9 | 103 | 20.3 |
|  |  |  |  | 349.0 | 3.00 | 91 | 10 | 27 | 22 |  |  |  |  |  |  |
| Flusilazole | Pos | 9.21 | 315.9 | 247.1 | 3.52 | 71 | 10 | 25 | 22 | 5 | 0.997 | 7.3 | 6.4 | 94 | 18.9 |
|  |  |  |  | 165.0 | 3.52 | 71 | 10 | 37 | 16 |  |  |  |  |  |  |
| Flutianil | Pos | 9.75 | 426.9 | 191.9 | 3.63 | 140 | 10 | 33 | 22 | 5 | 0.989 | 12.4 | 11.5 | 113 | 35.9 |
|  |  |  |  | 410.9 | 3.63 | 140 | 10 | 41 | 48 |  |  |  |  |  |  |
| Flutriafol | Pos | 6.25 | 301.9 | 70.0 | 7.18 | 36 | 10 | 45 | 8 | 5 | 0.997 | 11.3 | 12.4 | 94 | 25.6 |
|  |  |  |  | 123.0 | 7.18 | 36 | 10 | 39 | 14 |  |  |  |  |  |  |
| Fluxapyroxad | Pos | 7.93 | 382.0 | 361.9 | 3.71 | 1 | 10 | 23 | 28 | 5 | 0.997 | 9.7 | 10.0 | 93 | 23.9 |
|  |  |  |  | 234.0 | 3.71 | 1 | 10 | 33 | 18 |  |  |  |  |  |  |
| Forchlorfenuron | Pos | 6.49 | 248.0 | 129.0 | 6.83 | 1 | 10 | 25 | 14 | 5 | 0.998 | 10.4 | 11.5 | 78 | 48.7 |
|  |  |  |  | 92.9 | 6.83 | 1 | 10 | 49 | 10 |  |  |  |  |  |  |
| Formetanate hydrochloride | Pos | 3.14 | 222.0 | 165.0 | 17.57 | 41 | 10 | 21 | 20 | 5 | 0.999 | 13.2 | 12.4 | 85 | 40.0 |
|  |  |  |  | 120.0 | 17.57 | 41 | 10 | 37 | 14 |  |  |  |  |  |  |
| Fosthiazate | Pos | 5.98 | 283.9 | 228.0 | 6.56 | 31 | 10 | 15 | 20 | 5 | 0.996 | 7.4 | 6.9 | 87 | 29.9 |
|  |  |  |  | 103.9 | 6.56 | 31 | 10 | 27 | 14 |  |  |  |  |  |  |
| Haloxyfop | Pos | 9.29 | 361.8 | 316.0 | 3.78 | 106 | 10 | 25 | 28 | 5 | 0.991 | 14.3 | 13.2 | 92 | 32.8 |
|  |  |  |  | 288.0 | 3.78 | 106 | 10 | 37 | 28 |  |  |  |  |  |  |
| Haloxyfop Methyl | Pos | 10.68 | 375.9 | 288.8 | 6.53 | 71 | 10 | 23 | 30 | 5 | 0.989 | 6.2 | 7.0 | 108 | 20.2 |
|  |  |  |  | 287.9 | 6.44 | 71 | 10 | 35 | 28 |  |  |  |  |  |  |
| Hexaconazole | Pos | 9.93 | 313.9 | 70.0 | 3.28 | 46 | 10 | 49 | 8 | 5 | 0.989 | 10.5 | 11.4 | 115 | 36.6 |
|  |  |  |  | 158.9 | 3.28 | 46 | 10 | 43 | 16 |  |  |  |  |  |  |
| Hexaflumuron | Pos | 10.89 | 460.8 | 158.0 | 7.01 | 66 | 10 | 25 | 16 | 5 | 0.989 | 9.4 | 8.7 | 97 | 19.7 |
|  |  |  |  | 140.9 | 7.01 | 66 | 10 | 71 | 16 |  |  |  |  |  |  |
| Hexythiazox | Pos | 12.12 | 353.0 | 228.0 | 12.36 | 41 | 10 | 21 | 16 | 5 | 0.988 | 6.5 | 6.8 | 93 | 19.1 |
|  |  |  |  | 168.1 | 12.36 | 41 | 10 | 35 | 16 |  |  |  |  |  |  |
| Imazalil | Pos | 5.88 | 296.9 | 159.0 | 6.12 | 1 | 10 | 31 | 14 | 5 | 0.995 | 9.2 | 10.5 | 77 | 49.5 |
|  |  |  |  | 201.0 | 6.12 | 1 | 10 | 25 | 16 |  |  |  |  |  |  |
| Imidacloprid | Pos | 4.02 | 256.0 | 209.0 | 8.61 | 26 | 10 | 23 | 12 | 5 | 0.997 | 10.4 | 10.2 | 79 | 46.9 |
|  |  |  |  | 175.1 | 8.57 | 26 | 10 | 27 | 16 |  |  |  |  |  |  |
| Indoxacarb | Pos | 10.85 | 527.9 | 249.0 | 6.94 | 36 | 10 | 23 | 14 | 5 | 0.993 | 12.9 | 11.4 | 89 | 33.9 |
|  |  |  |  | 203.0 | 6.96 | 36 | 10 | 55 | 20 |  |  |  |  |  |  |
| Ioxynil | Neg | 6.66 | 369.7 | 126.8 | 6.62 | -60 | -10 | -40 | -13 | 5 | 0.997 | 7.2 | 7.8 | 76 | 50.1 |
|  |  |  |  | 214.9 | 6.62 | -60 | -10 | -44 | -11 |  |  |  |  |  |  |
| Iprodione | Pos | 8.97 | 329.9 | 245.1 | 3.05 | 16 | 10 | 23 | 14 | 5 | 0.989 | 14.8 | 13.5 | 116 | 43.6 |
|  |  |  |  | 288.0 | 3.07 | 16 | 10 | 21 | 22 |  |  |  |  |  |  |
| Iprovalicarb | Pos | 8.53 | 321.1 | 119.2 | 3.00 | 1 | 10 | 33 | 14 | 5 | 0.991 | 13.0 | 13.4 | 94 | 28.6 |
|  |  |  |  | 203.2 | 3.00 | 1 | 10 | 13 | 12 |  |  |  |  |  |  |
| Isocarbophos | Pos | 6.49 | 306.9 | 231.0 | 6.83 | 1 | 10 | 21 | 20 | 5 | 0.989 | 9.3 | 10.2 | 78 | 47.8 |
|  |  |  |  | 273.0 | 6.83 | 1 | 10 | 11 | 20 |  |  |  |  |  |  |
| Isofenfos methyl | Pos | 9.52 | 331.9 | 231.0 | 3.87 | 1 | 10 | 21 | 20 | 5 | 0.988 | 5.8 | 5.9 | 88 | 26.7 |
|  |  |  |  | 273.0 | 3.87 | 1 | 10 | 9 | 20 |  |  |  |  |  |  |
| Isofetamid | Pos | 8.65 | 359.9 | 210.0 | 3.00 | 31 | 10 | 15 | 20 | 5 | 0.991 | 14.2 | 13.8 | 98 | 28.7 |
|  |  |  |  | 125.0 | 3.00 | 31 | 10 | 39 | 14 |  |  |  |  |  |  |
| Isoprocarb | Pos | 6.23 | 211.0 | 194.0 | 7.13 | 1 | 10 | 11 | 20 | 5 | 0.989 | 10.2 | 10.1 | 84 | 37.9 |
|  |  |  |  | 95.0 | 7.13 | 1 | 10 | 25 | 12 |  |  |  |  |  |  |
| Isoprothiolane | Pos | 8.01 | 290.9 | 231.0 | 3.53 | 21 | 10 | 15 | 16 | 5 | 0.997 | 8.8 | 7.4 | 90 | 26.6 |
|  |  |  |  | 188.9 | 3.53 | 21 | 10 | 27 | 16 |  |  |  |  |  |  |
| Isoproturon | Pos | 6.41 | 206.8 | 72.0 | 7.13 | 66 | 10 | 23 | 10 | 5 | 0.996 | 13.8 | 12.4 | 83 | 43.8 |
|  |  |  |  | 165.1 | 7.13 | 66 | 10 | 21 | 10 |  |  |  |  |  |  |
| Kresoxim methyl | Pos | 9.44 | 313.9 | 267.1 | 3.99 | 31 | 10 | 11 | 18 | 5 | 0.994 | 6.6 | 7.4 | 91 | 22.3 |
|  |  |  |  | 222.1 | 3.99 | 31 | 10 | 23 | 12 |  |  |  |  |  |  |
| Lenacil | Pos | 6.48 | 235.1 | 153.1 | 6.87 | 71 | 10 | 23 | 8 | 5 | 0.996 | 12.3 | 11.2 | 81 | 45.3 |
|  |  |  |  | 135.9 | 6.87 | 71 | 10 | 41 | 16 |  |  |  |  |  |  |
| Linuron | Pos | 7.21 | 248.9 | 159.8 | 7.05 | 46 | 10 | 33 | 24 | 5 | 0.999 | 8.3 | 8.9 | 94 | 20.5 |
|  |  |  |  | 182.0 | 7.05 | 46 | 10 | 33 | 20 |  |  |  |  |  |  |
| Lufenuron | Neg | 11.86 | 508.9 | 338.7 | 9.12 | -5 | -10 | -20 | -39 | 5 | 0.989 | 10.7 | 11.4 | 100 | 21.4 |
|  |  |  |  | 325.7 | 9.12 | -5 | -10 | -26 | -35 |  |  |  |  |  |  |
| Malathion | Pos | 8.19 | 330.9 | 127.1 | 3.10 | 21 | 10 | 17 | 12 | 5 | 0.992 | 11.1 | 11.0 | 97 | 23.0 |
|  |  |  |  | 284.9 | 3.64 | 21 | 10 | 11 | 16 |  |  |  |  |  |  |
| Malathion-d10 | Pos | 7.89 | 340.9 | 132.1 | 3.86 | 36 | 10 | 19 | 14 | - | - | - | - | - | - |
|  |  |  |  | 100.1 | 3.81 | 36 | 10 | 35 | 12 |  |  |  |  |  |  |
| Mandipropamid | Pos | 7.88 | 411.9 | 356.1 | 3.91 | 51 | 10 | 15 | 30 | 5 | 0.994 | 12.9 | 11.7 | 95 | 27.7 |
|  |  |  |  | 328.1 | 3.86 | 51 | 10 | 35 | 28 |  |  |  |  |  |  |
| Matrine | Pos | 3.03 | 248.8 | 150.1 | 40.27 | 156 | 10 | 43 | 16 | 5 | 0.989 | 10.4 | 9.7 | 118 | 41.6 |
|  |  |  |  | 148.0 | 40.27 | 156 | 10 | 43 | 18 |  |  |  |  |  |  |
| Mebendazole | Pos | 6.21 | 295.9 | 264.0 | 7.09 | 111 | 10 | 31 | 24 | 5 | 0.996 | 8.5 | 7.8 | 79 | 45.3 |
|  |  |  |  | 105.0 | 7.07 | 111 | 10 | 45 | 12 |  |  |  |  |  |  |
| Melentrifluconazole | Pos | 9.05 | 415.0 | 119.1 | 3.30 | 81 | 10 | 21 | 14 | 5 | 0.988 | 14.5 | 13.8 | 119 | 47.8 |
|  |  |  |  | 91.2 | 3.32 | 81 | 10 | 89 | 10 |  |  |  |  |  |  |
| Mepanipyrim | Pos | 8.42 | 224.3 | 106.1 | 3.00 | 86 | 10 | 33 | 12 | 5 | 0.990 | 6.9 | 7.4 | 78 | 46.1 |
|  |  |  |  | 77.0 | 3.00 | 86 | 10 | 55 | 10 |  |  |  |  |  |  |
| Metaflumizone-E | Pos | 11.05 | 506.8 | 178.0 | 7.29 | 146 | 10 | 33 | 22 | 5 | 0.991 | 10.5 | 11.6 | 118 | 41.7 |
|  |  |  |  | 286.9 | 7.29 | 146 | 10 | 35 | 28 |  |  |  |  |  |  |
| Metaflumizone-Z | Pos | 11.78 | 506.8 | 178.0 | 8.95 | 116 | 10 | 33 | 20 | 5 | 0.989 | 6.2 | 6.5 | 111 | 25.3 |
|  |  |  |  | 287.0 | 8.91 | 116 | 10 | 35 | 22 |  |  |  |  |  |  |
| Metalaxyl | Pos | 6.58 | 280.0 | 90.9 | 6.62 | 1 | 10 | 55 | 32 | 5 | 0.989 | 11.8 | 11.4 | 101 | 23.7 |
|  |  |  |  | 77.1 | 6.64 | 1 | 10 | 147 | 10 |  |  |  |  |  |  |
| Metamitron | Pos | 4.24 | 202.8 | 175.0 | 9.88 | 1 | 10 | 23 | 18 | 5 | 0.988 | 14.2 | 13.2 | 112 | 37.2 |
|  |  |  |  | 174.1 | 9.91 | 1 | 10 | 23 | 16 |  |  |  |  |  |  |
| Metconazole | Pos | 10.02 | 320.0 | 70.1 | 3.15 | 31 | 10 | 53 | 8 | 5 | 0.989 | 14.6 | 12.4 | 98 | 29.5 |
|  |  |  |  | 125.1 | 3.15 | 31 | 10 | 53 | 12 |  |  |  |  |  |  |
| Methamidophos | Pos | 2.14 | 141.9 | 94.1 | 210.60 | 31 | 10 | 19 | 10 | 5 | 0.991 | 13.0 | 11.9 | 83 | 42.8 |
|  |  |  |  | 125.0 | 210.60 | 31 | 10 | 19 | 14 |  |  |  |  |  |  |
| Methidathion | Pos | 6.79 | 302.8 | 145.1 | 7.08 | 6 | 10 | 11 | 20 | 5 | 0.999 | 10.7 | 8.4 | 90 | 29.3 |
|  |  |  |  | 85.0 | 7.08 | 6 | 10 | 33 | 10 |  |  |  |  |  |  |
| Methiocarb | Pos | 7.45 | 226.0 | 169.1 | 5.70 | 21 | 10 | 15 | 14 | 5 | 0.997 | 11.3 | 10.4 | 91 | 28.9 |
|  |  |  |  | 121.0 | 5.70 | 21 | 10 | 27 | 14 |  |  |  |  |  |  |
| Methiocarb sulfone | Pos | 4.32 | 275.0 | 258.0 | 10.90 | 16 | 10 | 13 | 22 | 5 | 0.999 | 12.6 | 10.6 | 98 | 25.5 |
|  |  |  |  | 122.0 | 10.90 | 16 | 10 | 31 | 12 |  |  |  |  |  |  |
| Methiocarb sulfoxide | Pos | 4.16 | 241.9 | 185.0 | 9.70 | 41 | 10 | 21 | 16 | 5 | 0.998 | 7.5 | 9.4 | 85 | 33.5 |
|  |  |  |  | 170.0 | 9.70 | 41 | 10 | 31 | 10 |  |  |  |  |  |  |
| Methomyl | Pos | 3.66 | 163.0 | 88.0 | 7.10 | 16 | 10 | 13 | 12 | 5 | 0.989 | 14.1 | 13.2 | 86 | 39.7 |
|  |  |  |  | 106.0 | 7.10 | 16 | 10 | 15 | 14 |  |  |  |  |  |  |
| Methoxyfenozide | Pos | 8.24 | 369.1 | 313.1 | 3.00 | 21 | 10 | 11 | 26 | 5 | 0.996 | 10.2 | 9.4 | 88 | 31.5 |
|  |  |  |  | 149.1 | 3.00 | 21 | 10 | 23 | 16 |  |  |  |  |  |  |
| Metobromuron | Pos | 6.09 | 258.9 | 170.0 | 6.76 | 16 | 10 | 27 | 14 | 5 | 0.993 | 13.9 | 11.6 | 83 | 43.9 |
|  |  |  |  | 148.0 | 6.76 | 16 | 10 | 21 | 14 |  |  |  |  |  |  |
| Metolachlor | Pos | 8.99 | 284.0 | 252.1 | 3.10 | 46 | 10 | 23 | 22 | 5 | 0.993 | 9.8 | 7.4 | 87 | 32.6 |
|  |  |  |  | 176.1 | 3.10 | 46 | 10 | 37 | 18 |  |  |  |  |  |  |
| Metolcarb | Pos | 4.97 | 166.0 | 109.1 | 12.23 | 21 | 10 | 19 | 12 | 5 | 0.988 | 10.4 | 11.2 | 97 | 21.6 |
|  |  |  |  | 94.0 | 12.23 | 21 | 10 | 43 | 10 |  |  |  |  |  |  |
| Metrafenone | Pos | 10.32 | 408.9 | 209.0 | 4.48 | 46 | 10 | 21 | 20 | 5 | 0.991 | 10.8 | 11.4 | 97 | 22.4 |
|  |  |  |  | 227.0 | 4.48 | 46 | 10 | 29 | 14 |  |  |  |  |  |  |
| Monocrotophos | Pos | 3.78 | 224.0 | 193.0 | 7.36 | 26 | 10 | 11 | 10 | 5 | 0.999 | 11.5 | 9.6 | 86 | 36.2 |
|  |  |  |  | 127.1 | 7.36 | 26 | 10 | 21 | 12 |  |  |  |  |  |  |
| Monolinuron | Pos | 5.77 | 214.9 | 126.0 | 5.82 | 36 | 10 | 27 | 14 | 5 | 0.996 | 14.2 | 12.5 | 82 | 45.9 |
|  |  |  |  | 99.0 | 5.82 | 36 | 10 | 65 | 12 |  |  |  |  |  |  |
| Monuron | Pos | 5.12 | 199.0 | 72.1 | 9.26 | 26 | 10 | 21 | 8 | 5 | 0.998 | 9.9 | 9.4 | 101 | 19.9 |
|  |  |  |  | 125.9 | 9.26 | 26 | 10 | 35 | 14 |  |  |  |  |  |  |
| Myclobutanyl | Pos | 8.12 | 289.0 | 69.9 | 3.28 | 51 | 10 | 21 | 10 | 5 | 0.995 | 13.5 | 13.6 | 94 | 29.5 |
|  |  |  |  | 125.0 | 3.28 | 51 | 10 | 49 | 14 |  |  |  |  |  |  |
| Neburon | Pos | 9.21 | 274.9 | 88.1 | 3.52 | 66 | 10 | 21 | 12 | 5 | 0.996 | 12.0 | 12.4 | 88 | 33.9 |
|  |  |  |  | 114.1 | 3.52 | 66 | 10 | 21 | 14 |  |  |  |  |  |  |
| Nitenpyram | Pos | 3.47 | 271.0 | 237.1 | 7.68 | 76 | 10 | 27 | 14 | 5 | 0.990 | 11.4 | 9.4 | 78 | 49.6 |
|  |  |  |  | 126.0 | 7.68 | 76 | 10 | 37 | 14 |  |  |  |  |  |  |
| Novaluron | Pos | 11.09 | 492.9 | 158.0 | 7.61 | 66 | 10 | 27 | 20 | 5 | 0.989 | 11.9 | 10.2 | 113 | 35.2 |
|  |  |  |  | 141.1 | 7.52 | 66 | 10 | 27 | 26 |  |  |  |  |  |  |
| Ochratoxin A | Pos | 8.31 | 404.0 | 238.9 | 3.00 | 46 | 10 | 33 | 28 | 1 | 0.988 | 10.4 | 10.6 | 92 | 26.2 |
|  |  |  |  | 357.9 | 3.00 | 46 | 10 | 21 | 36 |  |  |  |  |  |  |
| Ochratoxin A 13C | Pos | 8.30 | 424.2 | 250.0 | 3.00 | 60 | 10 | 35 | 23 | - | - | - | - | - | - |
|  |  |  |  | 377.1 | 3.00 | 60 | 10 | 20 | 35 |  |  |  |  |  |  |
| Omethoate | Pos | 3.16 | 213.9 | 125.0 | 15.71 | 31 | 10 | 27 | 14 | 5 | 0.998 | 10.9 | 9.5 | 91 | 28.3 |
|  |  |  |  | 183.0 | 15.71 | 31 | 10 | 17 | 18 |  |  |  |  |  |  |
| Orthosulfamuron | Neg | 6.49 | 422.9 | 267.9 | 6.83 | -25 | -10 | -18 | -31 | 5 | 0.997 | 11.5 | 10.9 | 81 | 44.4 |
|  |  |  |  | 242.0 | 6.87 | -25 | -10 | -22 | -23 |  |  |  |  |  |  |
| Oxadiargyl | Pos | 10.09 | 340.9 | 223.0 | 3.34 | 76 | 10 | 25 | 42 | 5 | 0.988 | 13.2 | 13.6 | 97 | 27.1 |
|  |  |  |  | 229.9 | 3.29 | 76 | 10 | 33 | 30 |  |  |  |  |  |  |
| Oxadixyl | Pos | 4.86 | 279.0 | 219.1 | 13.07 | 21 | 10 | 17 | 12 | 5 | 0.997 | 12.7 | 12.6 | 98 | 25.7 |
|  |  |  |  | 132.2 | 13.07 | 21 | 10 | 43 | 14 |  |  |  |  |  |  |
| Oxamyl | Pos | 3.50 | 237.0 | 72.0 | 7.48 | 1 | 10 | 37 | 8 | 5 | 0.998 | 10.7 | 9.1 | 94 | 24.5 |
|  |  |  |  | 220.1 | 7.48 | 1 | 10 | 7 | 18 |  |  |  |  |  |  |
| Oxasulfuron | Pos | 5.13 | 407.0 | 150.0 | 9.15 | 1 | 10 | 15 | 22 | 5 | 0.992 | 11.4 | 11.6 | 93 | 26.8 |
|  |  |  |  | 107.1 | 9.15 | 1 | 10 | 27 | 18 |  |  |  |  |  |  |
| Oxathiapipronil | Pos | 8.17 | 539.9 | 500.0 | 3.14 | 146 | 10 | 35 | 20 | 5 | 0.998 | 9.5 | 7.4 | 100 | 19.0 |
|  |  |  |  | 522.0 | 3.14 | 146 | 10 | 37 | 22 |  |  |  |  |  |  |
| Oxfendazole | Pos | 5.05 | 316.0 | 159.1 | 10.41 | 111 | 10 | 43 | 16 | 5 | 0.993 | 9.2 | 9.1 | 78 | 47.7 |
|  |  |  |  | 191.1 | 10.41 | 111 | 10 | 29 | 22 |  |  |  |  |  |  |
| Oxydemeton-methyl | Pos | 3.61 | 247.0 | 168.9 | 7.20 | 31 | 10 | 19 | 20 | 5 | 0.994 | 14.8 | 13.4 | 93 | 32.7 |
|  |  |  |  | 108.9 | 7.20 | 31 | 10 | 31 | 12 |  |  |  |  |  |  |
| Paclobutrazol | Pos | 7.88 | 294.0 | 70.1 | 3.91 | 21 | 10 | 47 | 8 | 5 | 0.989 | 8.5 | 8.2 | 90 | 26.2 |
|  |  |  |  | 125.0 | 3.91 | 21 | 10 | 51 | 12 |  |  |  |  |  |  |
| Penconazole | Pos | 9.54 | 283.9 | 159.0 | 3.85 | 41 | 10 | 41 | 16 | 5 | 0.990 | 10.7 | 11.2 | 89 | 30.7 |
|  |  |  |  | 70.1 | 3.85 | 41 | 10 | 21 | 8 |  |  |  |  |  |  |
| Pencycuron | Pos | 10.41 | 329.0 | 125.0 | 5.08 | 96 | 10 | 31 | 14 | 5 | 0.988 | 7.3 | 7.6 | 94 | 18.9 |
|  |  |  |  | 89.1 | 5.08 | 96 | 10 | 91 | 10 |  |  |  |  |  |  |
| Pendimethalin | Pos | 12.09 | 282.2 | 212.0 | 11.57 | 1 | 10 | 17 | 22 | 5 | 0.989 | 12.4 | 11.8 | 93 | 28.5 |
|  |  |  |  | 194.2 | 11.47 | 1 | 10 | 25 | 16 |  |  |  |  |  |  |
| Penflufen | Pos | 9.53 | 318.0 | 234.1 | 3.85 | 81 | 10 | 23 | 28 | 5 | 0.995 | 5.8 | 8.4 | 90 | 23.1 |
|  |  |  |  | 141.0 | 3.85 | 81 | 10 | 21 | 26 |  |  |  |  |  |  |
| Penthiopyrad | Pos | 9.53 | 360.0 | 276.0 | 3.85 | 121 | 10 | 29 | 16 | 5 | 0.992 | 11.9 | 10.2 | 97 | 24.5 |
|  |  |  |  | 177.0 | 3.85 | 121 | 10 | 63 | 14 |  |  |  |  |  |  |
| Permethrin | Pos | 14.09 | 407.9 | 183.2 | 28.03 | 16 | 10 | 31 | 10 | 5 | 0.988 | 12.3 | 9.5 | 111 | 33.0 |
|  |  |  |  | 355.0 | 28.03 | 16 | 10 | 13 | 16 |  |  |  |  |  |  |
| Phenothrin | Pos | 13.99 | 351.0 | 183.0 | 26.09 | 1 | 10 | 31 | 22 | 5 | 0.989 | 11.6 | 10.4 | 95 | 25.3 |
|  |  |  |  | 249.1 | 28.03 | 1 | 10 | 25 | 24 |  |  |  |  |  |  |
| Phenthoate | Pos | 9.33 | 320.9 | 247.0 | 3.90 | 51 | 10 | 21 | 38 | 5 | 0.989 | 11.2 | 11.3 | 87 | 34.3 |
|  |  |  |  | 135.0 | 3.90 | 51 | 10 | 37 | 26 |  |  |  |  |  |  |
| Phosalone | Pos | 10.16 | 367.9 | 182.0 | 3.66 | 36 | 10 | 19 | 10 | 5 | 0.994 | 8.4 | 6.5 | 91 | 24.6 |
|  |  |  |  | 111.0 | 3.66 | 36 | 10 | 57 | 12 |  |  |  |  |  |  |
| Phosmet | Pos | 7.00 | 317.8 | 160.2 | 6.70 | 36 | 10 | 25 | 20 | 5 | 0.998 | 10.4 | 12.1 | 84 | 38.2 |
|  |  |  |  | 133.1 | 6.70 | 36 | 10 | 49 | 14 |  |  |  |  |  |  |
| Phoxim | Pos | 10.04 | 298.9 | 129.0 | 3.19 | 36 | 10 | 17 | 14 | 5 | 0.989 | 11.7 | 10.4 | 110 | 30.8 |
|  |  |  |  | 77.1 | 3.19 | 36 | 10 | 47 | 10 |  |  |  |  |  |  |
| Pirimicarb | Pos | 5.21 | 238.8 | 182.2 | 7.93 | 36 | 10 | 23 | 22 | 5 | 0.993 | 10.9 | 10.6 | 110 | 29.6 |
|  |  |  |  | 72.1 | 7.93 | 36 | 10 | 37 | 8 |  |  |  |  |  |  |
| Pirimiphos-methyl | Pos | 10.06 | 305.9 | 164.1 | 3.25 | 116 | 10 | 31 | 14 | 5 | 0.993 | 14.9 | 13.5 | 101 | 29.9 |
|  |  |  |  | 108.0 | 3.25 | 116 | 10 | 39 | 14 |  |  |  |  |  |  |
| Prochloraz | Pos | 10.18 | 375.9 | 308.0 | 3.78 | 1 | 10 | 17 | 28 | 5 | 0.991 | 6.9 | 7.4 | 85 | 33.0 |
|  |  |  |  | 265.9 | 3.78 | 1 | 10 | 23 | 22 |  |  |  |  |  |  |
| Procymidone | Pos | 8.38 | 283.9 | 256.1 | 3.00 | 101 | 10 | 25 | 14 | 5 | 0.988 | 10.4 | 11.2 | 108 | 26.2 |
|  |  |  |  | 145.1 | 3.07 | 101 | 10 | 55 | 12 |  |  |  |  |  |  |
| Profenofos | Pos | 11.29 | 372.8 | 302.8 | 8.70 | 61 | 10 | 25 | 24 | 5 | 0.992 | 11.5 | 10.3 | 100 | 23.0 |
|  |  |  |  | 344.9 | 8.70 | 61 | 10 | 19 | 24 |  |  |  |  |  |  |
| Promecarb | Pos | 7.75 | 208.0 | 151.2 | 4.67 | 36 | 10 | 13 | 10 | 5 | 0.993 | 10.2 | 11.1 | 88 | 31.5 |
|  |  |  |  | 109.1 | 4.67 | 36 | 10 | 23 | 12 |  |  |  |  |  |  |
| Prometryn | Pos | 7.77 | 242.0 | 158.0 | 4.54 | 76 | 10 | 33 | 16 | 5 | 0.998 | 14.8 | 12.6 | 81 | 48.2 |
|  |  |  |  | 200.2 | 4.54 | 76 | 10 | 27 | 12 |  |  |  |  |  |  |
| Propamocarb | Pos | 3.19 | 189.1 | 102.0 | 13.90 | 36 | 10 | 23 | 12 | 5 | 0.995 | 8.6 | 8.4 | 77 | 49.1 |
|  |  |  |  | 144.1 | 13.90 | 36 | 10 | 19 | 14 |  |  |  |  |  |  |
| Propaquizafop | Pos | 11.73 | 444.0 | 100.2 | 8.90 | 96 | 10 | 23 | 12 | 5 | 0.988 | 13.9 | 13.2 | 99 | 27.9 |
|  |  |  |  | 371.0 | 8.90 | 96 | 10 | 23 | 40 |  |  |  |  |  |  |
| Propargite | Pos | 12.57 | 368.0 | 231.2 | 21.68 | 31 | 10 | 15 | 22 | 5 | 0.991 | 14.1 | 13.5 | 116 | 42.7 |
|  |  |  |  | 175.1 | 21.68 | 31 | 10 | 23 | 18 |  |  |  |  |  |  |
| Propazine | Pos | 7.37 | 230.0 | 146.1 | 6.24 | 31 | 10 | 33 | 18 | 5 | 0.997 | 9.1 | 8.4 | 83 | 38.6 |
|  |  |  |  | 187.9 | 6.24 | 31 | 10 | 25 | 22 |  |  |  |  |  |  |
| Propiconazole | Pos | 9.98 | 341.9 | 158.9 | 3.17 | 61 | 10 | 41 | 24 | 5 | 0.988 | 6.4 | 6.8 | 95 | 16.2 |
|  |  |  |  | 123.1 | 3.17 | 61 | 10 | 77 | 14 |  |  |  |  |  |  |
| Propoxur | Pos | 5.28 | 209.9 | 111.0 | 7.02 | 36 | 10 | 21 | 12 | 5 | 0.994 | 10.8 | 9.2 | 91 | 28.1 |
|  |  |  |  | 168.1 | 7.02 | 36 | 10 | 13 | 14 |  |  |  |  |  |  |
| Propyzamide | Pos | 7.84 | 256.9 | 191.0 | 4.16 | 41 | 10 | 21 | 18 | 5 | 0.993 | 14.2 | 13.7 | 104 | 29.5 |
|  |  |  |  | 190.0 | 4.16 | 41 | 10 | 21 | 10 |  |  |  |  |  |  |
| Proquinazid | Pos | 12.92 | 372.9 | 331.0 | 22.94 | 41 | 10 | 23 | 36 | 5 | 0.989 | 13.9 | 13.5 | 86 | 39.5 |
|  |  |  |  | 288.9 | 23.04 | 41 | 10 | 33 | 24 |  |  |  |  |  |  |
| Prosulfocarb | Pos | 11.06 | 252.0 | 91.1 | 7.34 | 41 | 10 | 39 | 10 | 5 | 0.991 | 13.2 | 13.6 | 91 | 32.0 |
|  |  |  |  | 128.1 | 7.34 | 41 | 10 | 17 | 12 |  |  |  |  |  |  |
| Prothioconazole | Neg | 9.70 | 341.9 | 306.1 | 3.72 | -20 | -10 | -24 | -29 | 5 | 0.994 | 14.8 | 12.4 | 94 | 31.9 |
|  |  |  |  | 125.1 | 3.72 | -20 | -10 | -32 | -7 |  |  |  |  |  |  |
| Prothiofos | Pos | 13.37 | 344.9 | 240.9 | 25.75 | 1 | 10 | 25 | 22 | 5 | 0.988 | 10.8 | 8.5 | 115 | 37.0 |
|  |  |  |  | 269.0 | 25.75 | 1 | 10 | 17 | 26 |  |  |  |  |  |  |
| Pymetrozine | Pos | 3.19 | 218.0 | 105.0 | 13.90 | 36 | 10 | 27 | 12 | 5 | 0.997 | 10.4 | 8.6 | 77 | 50.5 |
|  |  |  |  | 78.0 | 13.90 | 36 | 10 | 57 | 10 |  |  |  |  |  |  |
| Pyraclostrobin | Pos | 10.15 | 387.9 | 194.1 | 3.61 | 26 | 10 | 17 | 12 | 5 | 0.996 | 11.5 | 10.2 | 94 | 25.9 |
|  |  |  |  | 163.0 | 3.61 | 26 | 10 | 35 | 16 |  |  |  |  |  |  |
| Pyridaben | Pos | 13.53 | 365.0 | 309.1 | 24.83 | 46 | 10 | 19 | 18 | 5 | 0.991 | 9.8 | 10.4 | 105 | 22.0 |
|  |  |  |  | 147.2 | 24.83 | 46 | 10 | 33 | 16 |  |  |  |  |  |  |
| Pyridalyl | Pos | 14.89 | 489.8 | 109.0 | 158.34 | 36 | 10 | 15 | 18 | 5 | 0.988 | 10.7 | 11.2 | 113 | 33.7 |
|  |  |  |  | 182.9 | 158.34 | 36 | 10 | 75 | 12 |  |  |  |  |  |  |
| Pyridaphenthion | Pos | 8.37 | 341.0 | 189.1 | 3.00 | 51 | 10 | 29 | 20 | 5 | 0.999 | 7.6 | 8.5 | 89 | 26.7 |
|  |  |  |  | 205.1 | 3.00 | 51 | 10 | 31 | 18 |  |  |  |  |  |  |
| Pyridate | Pos | 14.02 | 379.0 | 207.0 | 26.41 | 21 | 10 | 25 | 18 | 5 | 0.988 | 9.3 | 9.9 | 88 | 30.4 |
|  |  |  |  | 351.1 | 26.41 | 21 | 10 | 15 | 30 |  |  |  |  |  |  |
| Pyrimethanil | Pos | 7.06 | 200.0 | 107.0 | 6.82 | 101 | 10 | 33 | 12 | 5 | 0.997 | 10.3 | 9.4 | 114 | 34.8 |
|  |  |  |  | 183.0 | 6.82 | 101 | 10 | 33 | 10 |  |  |  |  |  |  |
| Pyriofenone | Pos | 10.33 | 366.0 | 209.0 | 4.53 | 116 | 10 | 41 | 12 | 5 | 0.995 | 13.2 | 11.5 | 102 | 26.7 |
|  |  |  |  | 184.0 | 4.53 | 116 | 10 | 17 | 24 |  |  |  |  |  |  |
| Pyriproxyfen | Pos | 11.90 | 322.0 | 96.1 | 9.51 | 36 | 10 | 21 | 12 | 5 | 0.988 | 11.5 | 10.2 | 95 | 25.1 |
|  |  |  |  | 185.1 | 9.56 | 36 | 10 | 33 | 18 |  |  |  |  |  |  |
| Quinalphos | Pos | 9.37 | 298.9 | 162.9 | 3.97 | 36 | 10 | 31 | 14 | 5 | 0.988 | 14.3 | 13.4 | 86 | 40.0 |
|  |  |  |  | 242.9 | 3.97 | 36 | 10 | 15 | 18 |  |  |  |  |  |  |
| Quinoclamine | Pos | 5.06 | 207.9 | 105.1 | 10.28 | 61 | 10 | 33 | 18 | 5 | 0.994 | 12.8 | 11.3 | 103 | 26.3 |
|  |  |  |  | 89.2 | 10.28 | 61 | 10 | 55 | 10 |  |  |  |  |  |  |
| Quinoxyfen | Pos | 12.17 | 307.6 | 197.0 | 14.15 | 146 | 10 | 45 | 18 | 5 | 0.993 | 13.2 | 9.4 | 87 | 37.1 |
|  |  |  |  | 162.0 | 14.15 | 146 | 10 | 61 | 16 |  |  |  |  |  |  |
| Quizalofop | Pos | 8.99 | 344.9 | 299.0 | 3.10 | 91 | 10 | 27 | 18 | 5 | 0.988 | 11.8 | 10.2 | 79 | 48.2 |
|  |  |  |  | 244.0 | 3.07 | 91 | 10 | 37 | 26 |  |  |  |  |  |  |
| Quizalofop-ethyl | Pos | 11.36 | 373.0 | 299.0 | 8.83 | 111 | 10 | 27 | 26 | 5 | 0.992 | 9.5 | 8.7 | 99 | 19.1 |
|  |  |  |  | 271.0 | 8.83 | 111 | 10 | 35 | 16 |  |  |  |  |  |  |
| Rotenone | Pos | 9.24 | 395.0 | 213.1 | 3.60 | 111 | 10 | 31 | 20 | 5 | 0.994 | 9.4 | 8.9 | 92 | 24.7 |
|  |  |  |  | 192.0 | 3.60 | 111 | 10 | 33 | 20 |  |  |  |  |  |  |
| Simazine | Pos | 5.35 | 202.0 | 132.1 | 6.62 | 91 | 10 | 27 | 12 | 5 | 0.997 | 10.4 | 11.2 | 90 | 28.9 |
|  |  |  |  | 124.2 | 6.62 | 91 | 10 | 27 | 14 |  |  |  |  |  |  |
| Spinetoram | Pos | 11.07 | 748.3 | 142.1 | 7.40 | 51 | 10 | 23 | 14 | 5 | 0.991 | 8.4 | 7.4 | 96 | 18.6 |
|  |  |  |  | 98.1 | 7.34 | 51 | 10 | 51 | 12 |  |  |  |  |  |  |
| Spinetoram A | Pos | 11.07 | 748.3 | 142.1 | 7.40 | 1 | 10 | 37 | 16 | 5 | 0.997 | 11.5 | 11.6 | 83 | 41.0 |
|  |  |  |  | 98.1 | 7.40 | 1 | 10 | 101 | 12 |  |  |  |  |  |  |
| Spinetoram B | Pos | 11.63 | 760.2 | 142.1 | 8.59 | 1 | 10 | 35 | 18 | 5 | 0.993 | 11.8 | 10.6 | 84 | 39.8 |
|  |  |  |  | 98.1 | 8.60 | 1 | 10 | 99 | 12 |  |  |  |  |  |  |
| Spinosad A | Pos | 10.29 | 732.3 | 142.1 | 4.32 | 6 | 10 | 35 | 18 | 5 | 0.998 | 9.7 | 8.4 | 78 | 48.1 |
|  |  |  |  | 98.0 | 4.24 | 6 | 10 | 99 | 12 |  |  |  |  |  |  |
| Spinosad D | Pos | 10.90 | 746.3 | 142.1 | 7.05 | 16 | 10 | 37 | 16 | 5 | 0.995 | 12.6 | 10.4 | 91 | 31.0 |
|  |  |  |  | 98.0 | 7.05 | 16 | 10 | 105 | 12 |  |  |  |  |  |  |
| Spirodiclofen | Pos | 13.05 | 410.9 | 313.0 | 23.55 | 36 | 10 | 17 | 30 | 5 | 0.988 | 7.9 | 7.4 | 120 | 43.0 |
|  |  |  |  | 71.1 | 23.55 | 36 | 10 | 35 | 10 |  |  |  |  |  |  |
| Spiromesifen | Pos | 12.65 | 371.0 | 273.1 | 22.46 | 31 | 10 | 17 | 14 | 5 | 0.989 | 12.4 | 10.2 | 102 | 25.1 |
|  |  |  |  | 255.1 | 22.46 | 31 | 10 | 33 | 14 |  |  |  |  |  |  |
| Spirotetramat | Pos | 8.76 | 374.0 | 302.2 | 3.00 | 21 | 10 | 25 | 24 | 5 | 0.996 | 11.8 | 11.6 | 92 | 28.5 |
|  |  |  |  | 330.2 | 3.00 | 21 | 10 | 21 | 26 |  |  |  |  |  |  |
| Spiroxamine | Pos | 7.36 | 298.1 | 144.2 | 6.34 | 1 | 10 | 29 | 14 | 5 | 0.989 | 7.3 | 8.9 | 77 | 48.3 |
|  |  |  |  | 100.2 | 6.34 | 1 | 10 | 45 | 12 |  |  |  |  |  |  |
| Sterigmatocystin | Pos | 8.73 | 325.0 | 281.0 | 3.00 | 76 | 10 | 49 | 34 | 1 | 0.988 | 13.9 | 12.4 | 88 | 36.7 |
|  |  |  |  | 309.9 | 3.00 | 76 | 10 | 35 | 30 |  |  |  |  |  |  |
| Sulfoxaflor | Pos | 4.28 | 294.6 | 173.9 | 10.30 | 41 | 10 | 27 | 20 | 5 | 0.998 | 11.5 | 9.8 | 85 | 37.8 |
|  |  |  |  | 154.0 | 10.30 | 41 | 10 | 47 | 18 |  |  |  |  |  |  |
| Tau-Fluvalinate | Pos | 13.69 | 502.9 | 208.0 | 24.54 | 51 | 10 | 17 | 24 | 5 | 0.989 | 6.8 | 7.9 | 113 | 29.3 |
|  |  |  |  | 180.6 | 24.54 | 51 | 10 | 49 | 20 |  |  |  |  |  |  |
| Tebuconazole | Pos | 9.56 | 308.0 | 70.1 | 3.87 | 71 | 10 | 53 | 8 | 5 | 0.989 | 12.7 | 13.2 | 92 | 30.0 |
|  |  |  |  | 125.0 | 3.87 | 71 | 10 | 53 | 16 |  |  |  |  |  |  |
| Tebufenozide | Pos | 9.32 | 353.1 | 297.1 | 3.87 | 16 | 10 | 13 | 24 | 5 | 0.994 | 10.9 | 11.2 | 90 | 29.6 |
|  |  |  |  | 133.1 | 3.87 | 16 | 10 | 27 | 16 |  |  |  |  |  |  |
| Tebufenpyrad | Pos | 11.67 | 334.1 | 145.0 | 8.68 | 131 | 10 | 37 | 14 | 5 | 0.988 | 8.4 | 8.1 | 95 | 19.6 |
|  |  |  |  | 147.1 | 8.68 | 131 | 10 | 35 | 10 |  |  |  |  |  |  |
| Teflubenzuron | Neg | 11.45 | 378.9 | 338.7 | 8.89 | -5 | -10 | -16 | -39 | 5 | 0.993 | 9.9 | 7.4 | 77 | 50.1 |
|  |  |  |  | 195.8 | 8.89 | -5 | -10 | -32 | -23 |  |  |  |  |  |  |
| Terbutryn | Pos | 7.94 | 242.1 | 186.1 | 3.70 | 41 | 10 | 27 | 20 | 5 | 0.998 | 12.4 | 9.4 | 84 | 40.5 |
|  |  |  |  | 91.1 | 3.74 | 41 | 10 | 37 | 10 |  |  |  |  |  |  |
| Terbutylazine | Pos | 7.61 | 230.0 | 174.1 | 5.25 | 1 | 10 | 23 | 10 | 5 | 0.997 | 11.3 | 10.9 | 85 | 37.6 |
|  |  |  |  | 103.9 | 5.41 | 1 | 10 | 45 | 12 |  |  |  |  |  |  |
| Tetraconazole | Pos | 8.64 | 371.9 | 159.0 | 3.00 | 11 | 10 | 41 | 16 | 5 | 0.991 | 14.7 | 13.8 | 100 | 29.4 |
|  |  |  |  | 123.0 | 3.00 | 11 | 10 | 87 | 14 |  |  |  |  |  |  |
| Tetramethrin | Pos | 11.57 | 332.0 | 164.0 | 8.70 | 31 | 10 | 35 | 18 | 5 | 0.988 | 14.3 | 13.6 | 94 | 31.0 |
|  |  |  |  | 135.0 | 8.70 | 31 | 10 | 25 | 16 |  |  |  |  |  |  |
| TFNA | Neg | 3.23 | 189.8 | 146.0 | 12.66 | -5 | -10 | -32 | -9 | 5 | 0.996 | 6.9 | 8.4 | 77 | 48.0 |
|  |  |  |  | 69.1 | 13.17 | -5 | -10 | -46 | -9 |  |  |  |  |  |  |
| TFNG | Neg | 3.44 | 246.9 | 182.9 | 8.04 | -10 | -10 | -18 | -21 | 5 | 0.995 | 12.7 | 12.8 | 79 | 49.1 |
|  |  |  |  | 163.0 | 8.04 | -10 | -10 | -24 | -19 |  |  |  |  |  |  |
| Thiabendazole | Pos | 4.04 | 201.9 | 175.1 | 8.76 | 121 | 10 | 37 | 10 | 5 | 0.999 | 13.1 | 13.0 | 79 | 49.5 |
|  |  |  |  | 131.1 | 8.76 | 121 | 10 | 45 | 14 |  |  |  |  |  |  |
| Thiacloprid | Pos | 4.49 | 252.9 | 126.0 | 13.57 | 51 | 10 | 29 | 14 | 5 | 0.998 | 10.5 | 10.9 | 91 | 27.7 |
|  |  |  |  | 90.0 | 13.57 | 51 | 10 | 53 | 10 |  |  |  |  |  |  |
| Thiamethoxam | Pos | 3.69 | 291.8 | 211.0 | 7.07 | 36 | 10 | 19 | 14 | 5 | 0.994 | 11.4 | 9.4 | 109 | 29.0 |
|  |  |  |  | 181.0 | 7.07 | 36 | 10 | 33 | 16 |  |  |  |  |  |  |
| Thiobencarb | Pos | 10.24 | 258.0 | 125.1 | 4.08 | 36 | 10 | 29 | 14 | 5 | 0.990 | 9.7 | 7.6 | 86 | 34.1 |
|  |  |  |  | 89.0 | 4.08 | 36 | 10 | 71 | 14 |  |  |  |  |  |  |
| Tolclofos methyl | Pos | 10.08 | 301.1 | 268.9 | 3.29 | 96 | 10 | 23 | 26 | 5 | 0.988 | 10.6 | 9.4 | 116 | 38.4 |
|  |  |  |  | 125.1 | 3.26 | 96 | 10 | 23 | 12 |  |  |  |  |  |  |
| Tolfenpyrad | Pos | 11.82 | 385.5 | 197.0 | 8.97 | 166 | 10 | 35 | 18 | 5 | 0.989 | 11.9 | 10.5 | 113 | 35.2 |
|  |  |  |  | 154.0 | 9.02 | 166 | 10 | 61 | 18 |  |  |  |  |  |  |
| Triadimefon | Pos | 8.25 | 293.9 | 197.0 | 3.00 | 31 | 10 | 21 | 12 | 5 | 0.996 | 13.5 | 13.8 | 88 | 36.1 |
|  |  |  |  | 225.0 | 3.00 | 31 | 10 | 19 | 18 |  |  |  |  |  |  |
| Triallate | Pos | 12.10 | 303.7 | 142.9 | 11.78 | 56 | 10 | 41 | 6 | 5 | 0.988 | 11.4 | 11.6 | 107 | 26.8 |
|  |  |  |  | 86.1 | 11.57 | 56 | 10 | 21 | 14 |  |  |  |  |  |  |
| Triazophos | Pos | 8.33 | 313.9 | 162.1 | 3.00 | 46 | 10 | 25 | 16 | 5 | 0.989 | 12.7 | 12.9 | 86 | 37.8 |
|  |  |  |  | 119.0 | 3.00 | 46 | 10 | 53 | 16 |  |  |  |  |  |  |
| Triclocarban | Pos | 10.59 | 314.8 | 127.0 | 6.08 | 91 | 10 | 45 | 14 | 5 | 0.989 | 9.6 | 8.6 | 93 | 23.8 |
|  |  |  |  | 162.0 | 6.08 | 91 | 10 | 27 | 14 |  |  |  |  |  |  |
| Tricyclazole | Pos | 4.81 | 190.0 | 163.0 | 13.30 | 106 | 10 | 31 | 14 | 5 | 0.993 | 7.4 | 8.1 | 77 | 48.3 |
|  |  |  |  | 136.0 | 13.30 | 106 | 10 | 39 | 14 |  |  |  |  |  |  |
| Trifloxystrobin | Pos | 10.84 | 409.0 | 186.1 | 6.93 | 36 | 10 | 25 | 20 | 5 | 0.991 | 11.4 | 10.9 | 101 | 22.9 |
|  |  |  |  | 145.0 | 6.93 | 36 | 10 | 69 | 16 |  |  |  |  |  |  |
| Triflumizole | Pos | 10.97 | 346.0 | 278.0 | 7.22 | 21 | 10 | 15 | 30 | 5 | 0.997 | 14.3 | 12.8 | 90 | 34.9 |
|  |  |  |  | 73.1 | 7.22 | 21 | 10 | 21 | 8 |  |  |  |  |  |  |
| Triflumuron | Neg | 10.02 | 356.9 | 153.9 | 3.15 | -45 | -10 | -18 | -17 | 5 | 0.989 | 14.7 | 11.7 | 97 | 30.0 |
|  |  |  |  | 84.9 | 3.15 | -45 | -10 | -60 | -9 |  |  |  |  |  |  |
| Trinexapac ethyl | Pos | 6.64 | 253.1 | 207.0 | 6.56 | 31 | 10 | 17 | 20 | 5 | 0.989 | 13.2 | 10.7 | 104 | 27.6 |
|  |  |  |  | 185.0 | 6.56 | 31 | 10 | 17 | 24 |  |  |  |  |  |  |
| Trinexapac_methyl | Pos | 5.69 | 239.1 | 207.0 | 5.77 | 31 | 10 | 17 | 26 | 5 | 0.989 | 10.8 | 11.2 | 91 | 28.1 |
|  |  |  |  | 69.0 | 5.77 | 31 | 10 | 31 | 8 |  |  |  |  |  |  |
| Triticonazole | Pos | 8.61 | 318.0 | 70.1 | 3.00 | 46 | 10 | 55 | 8 | 5 | 0.988 | 12.1 | 9.4 | 87 | 35.5 |
|  |  |  |  | 124.9 | 3.00 | 46 | 10 | 59 | 14 |  |  |  |  |  |  |
| Tritosulfuron | Pos | 6.88 | 445.9 | 195.0 | 7.00 | 26 | 10 | 19 | 14 | 5 | 0.989 | 9.0 | 10.2 | 85 | 35.0 |
|  |  |  |  | 221.0 | 6.96 | 26 | 10 | 25 | 10 |  |  |  |  |  |  |
| Valifenalate | Pos | 8.18 | 398.9 | 155.0 | 3.12 | 26 | 10 | 47 | 18 | 5 | 0.996 | 8.8 | 7.4 | 81 | 41.9 |
|  |  |  |  | 214.0 | 3.21 | 26 | 10 | 17 | 20 |  |  |  |  |  |  |
| XMC I | Pos | 5.84 | 180.0 | 123.0 | 5.94 | 31 | 10 | 17 | 18 | 5 | 0.996 | 10.5 | 10.2 | 88 | 31.9 |
|  |  |  |  | 95.0 | 5.94 | 31 | 10 | 29 | 10 |  |  |  |  |  |  |
| Zearalenone | Pos | 8.18 | 319.0 | 301.1 | 3.12 | 26 | 10 | 13 | 34 | 1 | 0.988 | 13.9 | 12.9 | 80 | 48.7 |
|  |  |  |  | 283.1 | 3.08 | 26 | 10 | 17 | 30 |  |  |  |  |  |  |
| Zoxamide | Pos | 9.69 | 335.9 | 186.9 | 3.74 | 56 | 10 | 31 | 18 | 5 | 0.944 | 13.2 | 10.4 | 85 | 40.0 |
|  |  |  |  | 159.0 | 3.74 | 56 | 10 | 57 | 18 |  |  |  |  |  |  |

Table S 3: Expanded measurement uncertainty calculated for groups of compounds with different LOQs, at two calibration levels

| **Group** | **LOQ (µg/kg)** | **U' at LOQ(%)** | **10*LOQ (µg/kg)** | **U' at 10*LOQ(%)** |
| --- | --- | --- | --- | --- |
| **Mycotoxins** | 1 | 27.8 | 5 | 19.3 |
| **Mycotoxins** | 100 | 24.4 | 1000 | 23.0 |
| **Pesticides** | 5 | 35.2 | 50 | 29.5 |

Table S 4: Recovery results for the QuEChERS and ASE methodologies applied to bee pollen

|  | **QuEChERS** | | **ASE** | |
| --- | --- | --- | --- | --- |
| **Compound** | **Recovery at 10µg/kg (%)** | **RSD (%)** | **Recovery at 10µg/kg (%)** | **RSD (%)** |
| 2,4-D | 75 | 6.4 | 109 | 5.0 |
| 8-Quinolinol | 79 | 14.6 | 112 | 14.0 |
| Acephate | 91 | 10.3 | 100 | 3.1 |
| Acetamiprid | 87 | 8.2 | 88 | 7.9 |
| Acrinathrin | 111 | 13.2 | 111 | 8.8 |
| Aflatoxin B1 | 103 | 10.2 | 117 | 9.1 |
| Alachlor | 84 | 3.6 | 98 | 7.5 |
| Albendazole | 83 | 8.9 | 113 | 5.8 |
| Aldicarb | 97 | 7.7 | 97 | 7.1 |
| Ametoctradin | 85 | 5.1 | 104 | 12.3 |
| Amisulbrom | 107 | 7.4 | 112 | 5.5 |
| Anilofos | 107 | 15.0 | 112 | 6.4 |
| Apicidin | 105 | 8.3 | 109 | 6.1 |
| Atrazine | 86 | 14.8 | 90 | 9.3 |
| Avermectin_B1a | 101 | 6.6 | 110 | 8.4 |
| Azinphos_methyl | 78 | 5.9 | 103 | 9.3 |
| Azinphos-ethyl | 77 | 7.3 | 109 | 5.5 |
| Azoxystrobin | 98 | 7.9 | 102 | 11.6 |
| BAC 10 | 83 | 8.7 | 103 | 8.6 |
| BAC 8 | 78 | 7.1 | 105 | 5.2 |
| Benalaxyl | 100 | 5.5 | 107 | 6.3 |
| Bendiocarb | 88 | 5.0 | 108 | 9.1 |
| Benzovindiflupyr | 96 | 11.8 | 101 | 11.1 |
| Bifenazate | 93 | 14.0 | 119 | 14.0 |
| Bifenazate diazene | 79 | 13.0 | 85 | 5.8 |
| Bifenthrin | 111 | 14.7 | 110 | 12.3 |
| Bitertanol | 108 | 9.1 | 106 | 6.9 |
| Bixafen | 87 | 7.4 | 104 | 6.5 |
| Boscalid | 92 | 8.8 | 103 | 4.5 |
| Bromacil | 83 | 14.0 | 106 | 6.1 |
| Bromuconazole | 107 | 8.2 | 108 | 5.1 |
| Bupirimate | 95 | 7.4 | 102 | 6.3 |
| Buprofezin | 88 | 9.2 | 102 | 3.7 |
| Butoxycarboxim | 99 | 3.6 | 104 | 5.9 |
| Carbaryl | 83 | 14.0 | 88 | 5.2 |
| Carbendazim | 79 | 13.7 | 89 | 3.6 |
| Chlorantraniliprole | 98 | 11.2 | 109 | 7.3 |
| Chlorbromuron | 84 | 8.4 | 101 | 4.8 |
| Chlorfenvinphos | 110 | 7.3 | 103 | 9.2 |
| Chlorfluazuron | 111 | 7.4 | 98 | 2.7 |
| Chloridazon | 80 | 7.8 | 106 | 5.8 |
| Chlorotoluron | 83 | 9.7 | 73 | 14.0 |
| Chloroxuron | 88 | 7.6 | 109 | 11.0 |
| Chlorpyrifos methyl | 93 | 6.1 | 95 | 6.9 |
| Chlorpyriphos | 106 | 8.1 | 113 | 12.0 |
| Chromafenozide | 105 | 5.6 | 100 | 8.4 |
| Clofentezine | 88 | 7.9 | 103 | 4.6 |
| Clomazone | 87 | 4.8 | 100 | 7.4 |
| Coumaphos | 91 | 3.9 | 104 | 6.8 |
| Cyantraniliprole | 107 | 3.3 | 117 | 13.9 |
| Cyazofamid | 115 | 6.6 | 108 | 9.4 |
| Cyflufenamid | 87 | 11.6 | 100 | 6.3 |
| Cyflumetofen | 112 | 3.8 | 100 | 4.6 |
| Cyhalofop-butyl | 114 | 7.7 | 102 | 2.9 |
| Cymoxanil | 91 | 13.5 | 95 | 11.7 |
| Cypermethrin | 94 | 3.3 | 102 | 9.9 |
| Cyproconazole | 104 | 9.9 | 103 | 6.7 |
| Cyprodinil | 76 | 11.8 | 97 | 4.9 |
| Dazomet | 78 | 6.9 | 108 | 6.2 |
| DEET | 87 | 4.1 | 106 | 5.3 |
| Demeton-S-methyl | 91 | 10.6 | 108 | 4.1 |
| Demeton-S-methylsulfone | 102 | 9.5 | 108 | 5.9 |
| Demeton-S-methylsulfoxide | 95 | 5.1 | 71 | 7.2 |
| Deoxynivalenol | 96 | 7.9 | 110 | 6.6 |
| Desethylterbuthylazine | 80 | 4.1 | 104 | 5.9 |
| Diazinon | 99 | 13.2 | 112 | 6.6 |
| Dichlorvos | 113 | 14.0 | 114 | 4.6 |
| Dicrotophos | 93 | 12.4 | 110 | 9.5 |
| Diethofencarb | 84 | 3.3 | 112 | 7.9 |
| Difenoconazole | 98 | 12.0 | 111 | 6.4 |
| Difenoxuron | 88 | 11.2 | 103 | 3.6 |
| Diflubenzuron | 113 | 5.1 | 117 | 14.2 |
| Dimethoate | 85 | 4.6 | 112 | 8.9 |
| Dimethomorph | 91 | 10.6 | 104 | 7.4 |
| Dimethylvinphos-Z | 97 | 5.5 | 85 | 8.9 |
| Diniconazole | 98 | 8.3 | 109 | 5.4 |
| Dinotefuran | 114 | 5.9 | 104 | 4.1 |
| Diuron | 84 | 14.2 | 81 | 12.3 |
| DMF | 108 | 7.4 | 103 | 10.9 |
| DMPF | 78 | 6.2 | 99 | 9.1 |
| Edifenphos | 93 | 8.5 | 105 | 14.7 |
| Emamectin B1a | 79 | 6.5 | 105 | 8.6 |
| Emamectin B1b | 102 | 5.5 | 119 | 9.3 |
| Enniatin A | 98 | 12.3 | 109 | 10.6 |
| Enniatin B | 95 | 7.2 | 110 | 8.5 |
| EPN | 119 | 8.8 | 106 | 7.4 |
| Epoxiconazole | 109 | 9.1 | 95 | 6.3 |
| Ethiofencarb | 88 | 5.5 | 109 | 10.4 |
| Ethion | 103 | 5.2 | 78 | 12.7 |
| Ethiprole | 91 | 7.4 | 79 | 10.1 |
| Ethirimol | 82 | 11.4 | 91 | 7.1 |
| Ethoprophos | 92 | 9.0 | 98 | 5.5 |
| Etofenprox | 104 | 7.3 | 100 | 11.6 |
| Etoxazole | 116 | 9.1 | 116 | 8.9 |
| Famoxadone | 93 | 12.8 | 109 | 9.7 |
| Fenamidone | 88 | 8.3 | 118 | 7.1 |
| Fenamiphos | 94 | 7.9 | 111 | 8.1 |
| Fenamiphos - sulfone | 91 | 12.0 | 73 | 14.7 |
| Fenamiphos - sulfoxide | 92 | 8.4 | 97 | 5.6 |
| Fenarimol | 89 | 4.7 | 76 | 14.6 |
| Fenazaquin | 93 | 7.9 | 108 | 5.8 |
| Fenbendazole | 79 | 6.9 | 77 | 14.9 |
| Fenbuconazole | 90 | 4.6 | 110 | 7.9 |
| fenhexamid | 95 | 5.9 | 105 | 6.9 |
| Fenobucarb | 87 | 9.6 | 108 | 7.4 |
| Fenoxycarb | 91 | 14.6 | 102 | 4.2 |
| Fenpicoxamid | 97 | 11.1 | 100 | 9.0 |
| Fenpropathrin | 112 | 4.6 | 107 | 8.2 |
| Fenpropidin | 75 | 5.8 | 105 | 7.2 |
| Fenpropimorph | 79 | 5.4 | 104 | 6.1 |
| Fenpyrazamine | 89 | 4.7 | 107 | 8.8 |
| Fenpyroximate-E | 114 | 6.2 | 104 | 12.0 |
| Fensulfothion | 89 | 12.8 | 106 | 3.9 |
| Fenthion | 109 | 6.8 | 110 | 5.5 |
| Fenthion sulfone | 94 | 6.5 | 105 | 4.5 |
| Fenthion sulfoxide | 84 | 5.6 | 103 | 10.6 |
| Fenuron | 89 | 12.4 | 109 | 9.4 |
| Fipronil | 112 | 6.1 | 108 | 7.1 |
| Flazasulfuron | 78 | 3.6 | 102 | 8.0 |
| Flonicamid | 106 | 7.1 | 107 | 9.5 |
| Florpyrauxifen_benzyl | 98 | 11.3 | 105 | 11.6 |
| Fluacrypyrim | 104 | 7.9 | 101 | 6.5 |
| Fluazifop | 85 | 14.0 | 110 | 7.6 |
| Flubendiamide | 93 | 7.8 | 74 | 14.8 |
| Flucythrinate | 113 | 6.8 | 103 | 6.2 |
| Fludioxonil | 84 | 7.3 | 108 | 6.3 |
| Flufenacet | 77 | 9.4 | 76 | 9.7 |
| Flufenoxuron | 114 | 12.8 | 113 | 5.1 |
| Fluometuron | 84 | 14.7 | 111 | 5.6 |
| Fluopicolide | 92 | 4.8 | 105 | 2.9 |
| Fluopyram | 92 | 4.7 | 104 | 7.9 |
| Flupyradifurone | 94 | 4.6 | 107 | 7.5 |
| Fluquinconazole | 103 | 8.9 | 112 | 9.3 |
| Flusilazole | 94 | 11.0 | 108 | 6.1 |
| Flutianil | 113 | 6.0 | 101 | 10.3 |
| Flutriafol | 94 | 13.9 | 81 | 13.7 |
| Fluxapyroxad | 93 | 8.2 | 107 | 7.2 |
| Forchlorfenuron | 78 | 13.7 | 73 | 13.9 |
| Formetanate hydrochloride | 85 | 7.3 | 98 | 14.0 |
| Fosthiazate | 87 | 12.4 | 117 | 14.6 |
| Haloxyfop | 92 | 3.1 | 97 | 6.1 |
| Haloxyfop Methyl | 108 | 7.3 | 99 | 4.3 |
| Hexaconazole | 115 | 14.9 | 91 | 11.4 |
| Hexaflumuron | 97 | 13.2 | 112 | 4.7 |
| Hexythiazox | 93 | 5.9 | 104 | 8.8 |
| Imazalil | 76 | 9.3 | 82 | 13.2 |
| Imidacloprid | 79 | 13.9 | 120 | 8.2 |
| Indoxacarb | 89 | 13.2 | 74 | 14.7 |
| Ioxynil | 75 | 4.6 | 99 | 13.5 |
| Iprodione | 116 | 14.0 | 80 | 14.6 |
| Iprovalicarb | 94 | 14.8 | 113 | 8.0 |
| Isocarbophos | 78 | 3.4 | 78 | 14.7 |
| Isofenfos methyl | 88 | 10.4 | 108 | 6.0 |
| Isofetamid | 98 | 4.1 | 105 | 8.4 |
| Isoprocarb | 84 | 12.0 | 105 | 7.2 |
| Isoprothiolane | 90 | 2.9 | 105 | 7.8 |
| Isoproturon | 83 | 8.2 | 104 | 5.8 |
| Kresoxim methyl | 91 | 14.7 | 106 | 11.3 |
| Lenacil | 81 | 5.6 | 105 | 7.6 |
| Linuron | 94 | 13.7 | 70 | 14.7 |
| Lufenuron | 100 | 11.5 | 104 | 8.5 |
| Malathion | 97 | 9.7 | 86 | 9.8 |
| Mandipropamid | 95 | 14.6 | 112 | 7.7 |
| Matrine | 118 | 8.5 | 103 | 2.3 |
| Mebendazole | 79 | 5.2 | 120 | 14.1 |
| Melentrifluconazole | 119 | 14.7 | 105 | 5.7 |
| Mepanipyrim | 78 | 7.3 | 102 | 3.8 |
| Metaflumizone-E | 118 | 8.1 | 103 | 5.7 |
| Metaflumizone-Z | 111 | 5.0 | 109 | 8.0 |
| Metalaxyl | 101 | 13.9 | 110 | 8.3 |
| Metamitron | 112 | 3.0 | 95 | 2.1 |
| Metconazole | 98 | 5.4 | 108 | 5.0 |
| Methamidophos | 83 | 3.3 | 119 | 5.2 |
| Methidathion | 90 | 5.6 | 118 | 13.0 |
| Methiocarb | 91 | 13.8 | 110 | 8.4 |
| Methiocarb sulfone | 98 | 4.0 | 109 | 9.1 |
| Methiocarb sulfoxide | 85 | 13.7 | 112 | 5.7 |
| Methomyl | 86 | 8.9 | 106 | 8.2 |
| Methoxyfenozide | 88 | 7.1 | 84 | 12.8 |
| Metobromuron | 83 | 14.6 | 72 | 14.8 |
| Metolachlor | 87 | 7.9 | 107 | 6.5 |
| Metolcarb | 97 | 6.1 | 105 | 4.8 |
| Metrafenone | 97 | 7.1 | 75 | 13.6 |
| Monocrotophos | 86 | 5.9 | 101 | 5.6 |
| Monolinuron | 82 | 11.7 | 77 | 7.8 |
| Monuron | 101 | 10.6 | 102 | 5.7 |
| Myclobutanyl | 94 | 6.4 | 78 | 12.2 |
| Neburon | 88 | 8.9 | 80 | 14.5 |
| Nitenpyram | 76 | 9.1 | 82 | 14.6 |
| Novaluron | 113 | 5.4 | 108 | 4.6 |
| Ochratoxin A | 92 | 6.1 | 107 | 4.6 |
| Omethoate | 91 | 13.2 | 89 | 14.9 |
| Orthosulfamuron | 81 | 13.7 | 76 | 14.3 |
| Oxadiargyl | 97 | 7.9 | 114 | 7.4 |
| Oxadixyl | 98 | 6.3 | 77 | 13.2 |
| Oxamyl | 94 | 6.5 | 100 | 7.3 |
| Oxasulfuron | 93 | 3.7 | 103 | 5.5 |
| Oxathiapipronil | 100 | 14.0 | 102 | 8.7 |
| Oxfendazole | 78 | 6.2 | 107 | 6.0 |
| Oxydemeton-methyl | 93 | 4.1 | 107 | 3.3 |
| Paclobutrazol | 90 | 5.4 | 106 | 5.2 |
| Penconazole | 89 | 8.2 | 88 | 7.3 |
| Pencycuron | 94 | 6.2 | 101 | 7.3 |
| Pendimethalin | 93 | 3.3 | 104 | 5.9 |
| Penflufen | 90 | 6.3 | 105 | 5.1 |
| Penthiopyrad | 97 | 10.7 | 107 | 7.9 |
| Permethrin | 111 | 10.3 | 99 | 3.0 |
| Phenothrin | 95 | 6.1 | 108 | 4.4 |
| Phenthoate | 87 | 8.4 | 105 | 4.3 |
| Phosalone | 91 | 14.5 | 112 | 10.7 |
| Phosmet | 84 | 13.7 | 72 | 12.4 |
| Phoxim | 110 | 7.9 | 98 | 3.6 |
| Pirimicarb | 110 | 7.2 | 107 | 7.2 |
| Pirimiphos-methyl | 101 | 6.4 | 106 | 5.9 |
| Prochloraz | 85 | 14.6 | 84 | 14.8 |
| Procymidone | 108 | 4.3 | 105 | 5.0 |
| Profenofos | 100 | 2.9 | 100 | 6.2 |
| Promecarb | 88 | 7.6 | 118 | 5.5 |
| Prometryn | 79 | 12.3 | 112 | 6.8 |
| Propamocarb | 77 | 5.1 | 106 | 5.6 |
| Propaquizafop | 99 | 12.8 | 98 | 7.3 |
| Propargite | 116 | 7.4 | 101 | 8.5 |
| Propazine | 83 | 4.5 | 104 | 8.5 |
| Propiconazole | 95 | 8.9 | 102 | 8.1 |
| Propoxur | 91 | 14.9 | 74 | 13.2 |
| Propyzamide | 104 | 5.8 | 111 | 7.3 |
| Proquinazid | 86 | 8.5 | 100 | 7.7 |
| Prosulfocarb | 91 | 14.3 | 86 | 5.0 |
| Prothioconazole | 94 | 9.3 | 116 | 3.3 |
| Prothiofos | 115 | 5.5 | 93 | 9.4 |
| Pymetrozine | 77 | 6.1 | 103 | 6.0 |
| Pyraclostrobin | 94 | 8.4 | 109 | 6.9 |
| Pyridaben | 105 | 6.4 | 94 | 4.7 |
| Pyridalyl | 113 | 5.6 | 109 | 5.4 |
| Pyridaphenthion | 89 | 5.5 | 114 | 14.6 |
| Pyridate | 88 | 2.8 | 94 | 6.4 |
| Pyrimethanil | 114 | 7.4 | 116 | 13.9 |
| Pyriofenone | 102 | 8.0 | 108 | 5.4 |
| Pyriproxyfen | 95 | 7.1 | 118 | 9.7 |
| Quinalphos | 86 | 12.0 | 110 | 4.4 |
| Quinoclamine | 103 | 14.6 | 116 | 14.0 |
| Quinoxyfen | 87 | 14.7 | 74 | 5.8 |
| Quizalofop | 79 | 6.4 | 76 | 12.0 |
| Quizalofop-ethyl | 99 | 12.2 | 74 | 13.7 |
| Rotenone | 92 | 4.3 | 96 | 6.4 |
| Simazine | 90 | 9.3 | 107 | 9.1 |
| Spinetoram | 96 | 5.2 | 98 | 5.6 |
| Spinetoram A | 83 | 7.1 | 92 | 13.7 |
| Spinetoram B | 84 | 5.2 | 88 | 13.5 |
| Spinosad A | 76 | 5.8 | 117 | 9.3 |
| Spinosad D | 91 | 14.6 | 118 | 14.3 |
| Spirodiclofen | 120 | 9.1 | 92 | 4.7 |
| Spiromesifen | 102 | 10.2 | 110 | 11.2 |
| Spirotetramat | 92 | 8.9 | 119 | 8.2 |
| Spiroxamine | 77 | 4.6 | 102 | 6.0 |
| Sterigmatocystin | 88 | 5.1 | 107 | 6.2 |
| Sulfoxaflor | 85 | 14.6 | 79 | 13.7 |
| Tau-Fluvalinate | 113 | 13.6 | 72 | 14.7 |
| Tebuconazole | 92 | 8.1 | 106 | 11.5 |
| Tebufenozide | 90 | 12.3 | 89 | 13.9 |
| Tebufenpyrad | 95 | 14.7 | 110 | 4.5 |
| Teflubenzuron | 77 | 4.7 | 76 | 13.7 |
| Terbutryn | 84 | 5.9 | 107 | 7.2 |
| Terbutylazine | 85 | 14.7 | 111 | 12.8 |
| Tetraconazole | 100 | 7.1 | 92 | 4.6 |
| Tetramethrin | 94 | 14.1 | 88 | 12.8 |
| TFNA | 75 | 13.8 | 74 | 5.1 |
| TFNG | 79 | 5.7 | 109 | 8.3 |
| Thiabendazole | 79 | 7.3 | 97 | 9.1 |
| Thiacloprid | 91 | 4.6 | 75 | 13.7 |
| Thiamethoxam | 109 | 12.3 | 103 | 4.7 |
| Thiobencarb | 86 | 5.4 | 107 | 9.6 |
| Tolclofos methyl | 116 | 5.0 | 106 | 7.1 |
| Tolfenpyrad | 113 | 9.3 | 86 | 13.2 |
| Triadimefon | 88 | 12.8 | 90 | 14.8 |
| Triallate | 107 | 13.9 | 81 | 14.9 |
| Triazophos | 86 | 5.1 | 111 | 4.0 |
| Triclocarban | 93 | 14.6 | 112 | 8.1 |
| Tricyclazole | 77 | 14.8 | 91 | 14.9 |
| Trifloxystrobin | 101 | 14.3 | 72 | 13.8 |
| Triflumizole | 90 | 3.3 | 79 | 13.2 |
| Triflumuron | 97 | 6.1 | 78 | 13.8 |
| Trinexapac ethyl | 104 | 14.6 | 89 | 12.7 |
| Trinexapac_methyl | 91 | 8.9 | 114 | 7.9 |
| Triticonazole | 87 | 6.3 | 99 | 9.1 |
| Tritosulfuron | 85 | 4.9 | 79 | 11.9 |
| Valifenalate | 81 | 4.5 | 101 | 11.2 |
| XMC I | 88 | 11.2 | 100 | 11.8 |
| Zearalenone | 80 | 14.8 | 110 | 12.4 |
| Zoxamide | 85 | 6.4 | 113 | 10.2 |

Table S 5: LC-MS/MS results obtained from the analysis of real bee pollen samples SL-01 to SP-14

| **Compound** | **Class** | **Number of positive samples** | **Positive samples [ID(µg/kg)]** | **Median concentration (µg/kg)** |
| --- | --- | --- | --- | --- |
| Ochratoxin A | Mycotoxin | 8 | SL-01 (1.07); SL-03 (1.06); SL-04 (1.08); SL-05 (3.22±0.44); SL-08 (0.99); SL-15 (1.05); SL-17 (1.08); SP-03 (1.03) | 1.32 |
| DMF | Acaricide - Vet | 6 | SP-01 (17.23±2.02); SP-04 (15.65±2.52); SP-08 (15.39±2.29); SP-10 (57.94±9.05); SP-12 (32.82±4.54); SP-13 (29.81±3.42) | 23.52 |
| Hexaconazole | Fungicide | 5 | SL-03 (24.72±3.64); SL-16 (5.89); SP-07 (13.42±1.12); SP-10 (10.04±1.36); SP-14 (15.59±2.48) | 13.42 |
| Spiroxamine | Fungicide | 5 | SL-03 (5.10); SL-15 (24.29±2.72); SL-20 (9.00); SP-10 (100.20±15.27); SP-12 (11.50±0.94) | 11.50 |
| Acrinathrin | Acaricide - Vet | 3 | SL-09 (19.04±2.94); SP-05 (7.02); SP-14 (9.99±1.14) | 9.99 |
| DMPF | Acaricide - Vet | 3 | SL-08 (24.68±4.12); SP-10 (11.86±1.68); SP-12 (5.72) | 11.87 |
| Tau-Fluvalinate | Acaricide - Vet | 3 | SP-10 (11.98±0.44); SP-11 (58.82±8.21); SP-13 (20.16±1.62); | 20.16 |
| Triticonazole | Fungicide | 3 | SL-08 (9.35±0.44); SL-10 (7.50); SL-14 (7.37) | 7.50 |
| Azoxystrobin | Fungicide | 2 | SL-17 (271.98±48.14); SP-14(14.81±2.02) | 143.40 |
| Bifenthrin | Insecticide | 2 | SL-10 (7.13); SL-15(9.59±1.01) | 8.36 |
| Cyhalofop-butyl | Herbicide | 2 | SL-02 (98.52±14.57); SL-07 (85.85±9.91) | 92.19 |
| Fluazifop | Herbicide | 2 | SL-05 (52.31±7.01); SP-14 (110.43±11.34) | 81.37 |
| Flucythrinate | Insecticide | 2 | SL-06 (5.47); SL-09 (5.84) | 5.66 |
| Methiocarb sulfone | Insecticide | 2 | SL-01 (8.32); SP-01 (5.13) | 6.72 |
| Quinoclamine | Herbicide | 2 | SL-06 (19.38±2.01); SL-11 (66.11±11.32) | 42.75 |
| Tebuconazole | Fungicide | 2 | SL-05 (6.61); SP-14 (16.31±2.10) | 11.46 |
| Tebufenpyrad | Insecticide | 2 | SL-02 (7.31); SL-05 (8.10) | 7.70 |
| Acetamiprid | Insecticide | 1 | SP-14 (6.18) | 6.18 |
| Azinphos-ethyl | Insecticide | 1 | SL-04 (8.24) | 8.24 |
| Boscalid | Fungicide | 1 | SP-14 (14.56±1.12) | 14.56 |
| Carbendazim | Fungicide | 1 | SP-14 (6.54) | 6.54 |
| Coumaphos | Acaricide - Vet | 1 | SP-11 (7.18) | 7.18 |
| Cyprodinil | Fungicide | 1 | SL-14 (15.82±1.69) | 15.83 |
| Dimethomorph | Fungicide | 1 | SL-15 (6.35) | 6.36 |
| Fenarimol | Fungicide | 1 | SL-14 (20.63±2.32) | 20.36 |
| Flonicamid | Insecticide | 1 | SL-17 (68.39±8.62) | 68.39 |
| Fludioxonil | Fungicide | 1 | SP-14 (5.28) | 5.28 |
| Fluopyram | Fungicide | 1 | SL-05 (19.81±2.56) | 19.81 |
| Haloxyfop | Herbicide | 1 | SP-09 (5.64) | 5.65 |
| Iprovalicarb | Fungicide | 1 | SP-10 (37.23±6.06) | 37.23 |
| Metamitron | Herbicide | 1 | SP-14 (5.59) | 5.59 |
| Metconazole | Fungicide | 1 | SL-02 (117.93±21.01) | 117.93 |
| Metolachlor | Herbicide | 1 | SL-13 (9.95±0.76) | 9.95 |
| Oxadiargyl | Herbicide | 1 | SL-18 (14.46±2.28) | 14.46 |
| Pendimethalin | Herbicide | 1 | SL-05 (15.38±1.94) | 15.38 |
| Propyzamide | Herbicide | 1 | SL-06 (7.78) | 7.78 |
| Pyraclostrobin | Fungicide | 1 | SP-14 (16.85±2.61) | 16.85 |
| Pyrimethanil | Fungicide | 1 | SL-17 (115.84±20.14) | 115.84 |
| Quizalofop | Herbicide | 1 | SP-14 (14.59±2.74) | 14.59 |
| Terbutylazine | Herbicide | 1 | SL-13 (5.11); | 5.11 |
| Thiacloprid | Insecticide | 1 | SP-14 (6.01) | 6.01 |
| Tolfenpyrad | Insecticide | 1 | SP-11 (5.11) | 5.11 |
